# Orchard and storage-dependent metabolic and transcriptional regulation of superficial scald in ‘Packham’s Triumph’ pears

**DOI:** 10.3389/fpls.2026.1885958

**Published:** 2026-08-27

**Authors:** Hnin Phyu Lwin, Jingi Yoo, Huiting Zhang, Heidi Hargarten, Loren Honaas, David R. Rudell, Bruno G. Defilippi, Nilo Mejía, Carolina A. Torres

**Affiliations:** 1 Department of Horticulture, Tree Fruit Research and Extension Center, Washington State University, Wenatchee, WA, United States; 2 Department of Horticulture, Washington State University, Pullman, WA, United States; 3 USDA-ARS, Physiology and Pathology of Tree Fruits Research, Wenatchee, WA, United States; 4 Instituto de Investigaciones Agropecuarias (INIA -Chile), La Platina Research Centre, Santiago, Chile

**Keywords:** controlled atmosphere, gene expression, metabolomic, pear, stepwise cooling, superficial scald

## Abstract

Superficial scald (SS), a chilling-induced peel disorder, limits the storability and marketability of cold-stored ‘Packham’s Triumph’ pears. This study combined gene expression and non-polar metabolomic profiling to elucidate orchard- and storage-specific mechanisms regulating SS development. Pears were sourced from three commercial orchards and stored under regular atmosphere (RA), controlled atmosphere (CA), or stepwise cooling (SWC). Controlled atmosphere (CA) consistently provided the greatest suppression of SS, whereas the effectiveness of stepwise cooling (SWC) varied among orchards. Gene expression analysis showed that storage atmosphere regulated stress- and senescence-related genes, with antioxidant-associated genes (GST and GPx) generally enhanced under CA and SWC, while lipid degradation- and senescence-associated genes (LPS, LOX, and SGR) exhibited storage- and orchard-dependent responses. Metabolomic analyses revealed coordinated changes in farnesyl conjugates, hydroxycinnamoyl acyl esters, diacylglycerols, triacylglycerols, phytosterol conjugates, and sphingolipids under scald-suppressive storage conditions. Correlation network analysis further identified coordinated associations between stress-responsive genes, particularly 9-cis-epoxycarotenoid dioxygenase (NCED), and metabolites involved in membrane lipid remodeling and secondary metabolism. These findings improve our understanding of the molecular basis of superficial scald in pear and identify candidate gene and metabolite biomarkers that could support orchard-specific scald risk assessment and the development of targeted postharvest storage strategies.

## 1 Introduction

Superficial scald (SS) is a postharvest physiological disorder that significantly impacts the quality and marketability of apples (*Malus domestica* Borkh.) and pears (*Pyrus communis* L.), particularly in long-term cold-stored fruit. This disorder manifests as irregular brown to black patches on the fruit peel, typically developing after 2–4 months of storage at 0–1 °C and worsening during subsequent shelf life, leading to significant economic losses and reduced marketability (Populin et al., 2023; Torres and Sepulveda, 2023). Among pear cultivars, ‘Packham’s Triumph’ is notably susceptible to SS, leading to substantial economic losses, especially in major exporting countries such as Chile and Argentina, where pears are stored for long-term to meet global market demands (Candan and Calvo, 2016; Torres et al., 2021).

SS has traditionally been associated with oxidative stress and the oxidation of α-farnesene, a sesquiterpene synthesized in the fruit peel. In apple fruit, the accumulation and subsequent oxidation of α-farnesene and its derivatives, particularly conjugated trienols (CTols), have been strongly linked to scald development (Whitaker, 2004; Lu et al., 2013; Donadel et al., 2023). Accordingly, storage strategies that suppress oxidative metabolism, including controlled atmosphere (CA), dynamic controlled atmosphere (DCA), and low-oxygen storage systems, effectively reduce scald incidence by limiting α-farnesene oxidation and associated oxidative damage (Watkins et al., 2000; Rudell et al., 2006; Larrigaudière et al., 2019; Populin et al., 2023; Botes et al., 2024). However, recent transcriptomic and metabolomic studies indicate that superficial scald is not solely driven by α-farnesene oxidation, but rather reflects a complex network involving membrane lipid remodeling, oxidative stress, antioxidant defenses, hormone signaling, and stress-response pathways during prolonged cold storage (Giné-Bordonaba et al., 2020; He et al., 2022a, 2022b; Vittani et al., 2023).

In contrast to apples, our understanding of SS in pears is still somewhat limited. Previous research has reported inconsistent findings regarding the relationship between α-farnesene accumulation and the susceptibility of different pear cultivars to scald, suggesting that other factors may be involved in the disorder’s development (Rudell et al., 2009; Gapper et al., 2017). Recent studies have identified a variety of factors that may contribute to pear scald, including lipid peroxidation, antioxidant metabolism, ethylene signaling, and the regulation of stress-responsive genes (Zhou et al., 2017; He et al., 2022a; Niu et al., 2024). These insights indicate that scald in pears likely arises from a complex interplay among oxidative damage, membrane remodeling, and aging processes, rather than from a single biochemical mechanism.

Preharvest conditions in the orchard can greatly affect SS development in fruits. Factors such as fruit maturity, orchard microclimate, and thermal history affect storage behavior (Lurie and Watkins, 2012; Torres et al., 2021) by influencing antioxidant capacity and stress tolerance (Watkins et al., 2000). In pears, the impact of harvest maturity is less consistent, with some studies reporting increased (Torres et al., 2021), decreased (Calvo et al., 2015), or no effect (Moggia et al., 2005) on disorder development. Additionally, low preharvest night temperatures (<10 °C) have been associated with reduced SS in apples (Thomai et al., 1998), while in pears, a predictive model based on accumulated cold units prior to harvest was developed for ‘d’Anjou’ (Ma et al., 2001). Packham’s Triumph pears highlighted significant orchard-related differences in scald incidence, suggesting that the fruit’s physiological condition at harvest is crucial for its susceptibility (Torres et al., 2021). Despite these insights, the molecular and metabolic reasons behind these orchard-specific responses remain unclear.

Metabolite profiling and targeted gene expression analysis are complementary methodologies for elucidating the biochemical pathways underlying the development of postharvest disorders. In apple cultivars, non-polar metabolite profiling has identified significant correlations between susceptibility to SS and alterations in triterpenoids, sterol conjugates, and lipid-derived metabolites (Rudell et al., 2009; Busatto et al., 2014; Leisso et al., 2016). Concurrently, targeted gene expression analyses have underscored the involvement of genes associated with oxidative stress responses, lipid metabolism, chlorophyll degradation, and hormonal regulation throughout the progression of SS (Leisso et al., 2016; Busatto et al., 2021; Cainelli et al., 2021; Honaas et al., 2021). Nevertheless, comparable integrative studies focusing on pears remain limited.

In this study, we investigated orchard- and storage-dependent variation in superficial scald development in ‘Packham’s Triumph’ pears stored under regular atmosphere (RA), controlled atmosphere (CA), and stepwise cooling (SWC) conditions. We hypothesized that orchard origin influences the transcriptional and metabolic responses of pear fruit to different storage conditions, resulting in differences in superficial scald susceptibility. To test this hypothesis, we combined targeted gene expression analysis with non-polar metabolite profiling to identify key genes and metabolites associated with orchard- and storage-dependent variation in superficial scald development.

## 2 Materials and methods

### 2.1 Fruit source and tissue collection

Pears (*Pyrus communis* L.) cv ‘Packham’s Triumph’ were harvested from three commercial orchards, named “Agrofruta” (Orchard 1; 35°35’12”S, 71°28’56”W), “Pirhuin 3” (Orchard 2; 35° 2’46”S, 71°14’43”W), and “Talcarehue” (Orchard 3; 34°38’16”S, 70°53’11”W) located in the main Chilean pear-growing region, characterize by a Mediterranean climate. All orchards were managed conventionally with standard commercial practices. Preharvest environmental conditions were characterized based on previously published data from the same orchards and seasons (Torres et al., 2021). Growing degree-days (GDA; base 10 °C) from full bloom to harvest and chill accumulation (HL10; hours <10 °C during the 45 d prior to harvest) were calculated using orchard-specific weather station data. In the present study, fruit were harvested one week before the commercial harvest date (120–132 days after full bloom), corresponding to the early maturity stage (H1) defined in Torres et al. (2021), where maturity was assessed by flesh firmness and starch index. Three postharvest storage conditions were evaluated in fruit from three orchards. These storage conditions were regular atmosphere (RA at -0.5 °C and >90%RH), controlled atmosphere (CA; O_2_: 2.0 kPa, CO_2_: 1.0 kPa, -0.5 °C, >90% RH) and stepwise cooling (SWC, 1 week at 5 °C, 1 week at 3 °C, 1 week at 2 °C and 1 week at -0.5 °C, >90%RH). Peel (fleshless) was collected from the equatorial region of five pears per replicate (n = 20; four replicates). These samples were immediately flash-frozen in liquid nitrogen and stored for molecular and metabolite analyses.

### 2.2 Fruit maturity assessment and superficial scald incidence evaluation

Fruit maturity was evaluated using flesh firmness (N), soluble solids content (SSC), starch index (SI, scale 1-6), and *I*_AD_ (0-2.2). Flesh firmness was measured on two opposite sides of each fruit using an 8 mm probe attached to a texture analyzer (GS14, GÜSS Manufacturing, South Africa), and values were expressed in Newtons (N). SSC was determined from extracted juice using a digital refractometer (PAL-1, Atago Inc., USA) and expressed as °Brix. The *I*_AD_ value, an indicator of chlorophyll content, was measured using a handheld non-destructive Delta Absorbance (DA) meter (Sinteleia, Italy). Fruit maturity was evaluated using four biological replicates of five fruit each (n = 20).

SS incidence was assessed after 140 and 180 days of cold storage, followed by a 14-day shelf-life period at 20 °C. Disorder incidence rate was visually evaluated and expressed as the percentage of affected fruit. Assessments were conducted using five replicates of 100 fruit per treatment and sampling time.

### 2.3 RNA extraction and quality assessment

RNA was extracted from the ground frozen tissue using a CATB/Chloroform protocol modified for use on pome fruit tissue in the postharvest period (Honaas and Kahn, 2017). Extracted RNA was analyzed for purity using the NanodropOne (Thermo Fisher Scientific, Waltham, MA USA) for integrity on the Agilent Bioanalyzer (Agilent, Santa Clara, CA USA, Agilent-RNA Pico Kit, Cat#: 5067-1513), and quantity using the Invitrogen™ Qubit™3 (Thermo Fisher Scientific, Waltham, MA USA, Qubit™ RNA HS Assay Kit, Cat#: Q32852). High quality total RNA was used for downstream qPCR (A260/A280 ≈ 2.0, RNA Integrity Number (RIN) ≥ 8.0).

### 2.4 Reference transcriptome assembly

RNA-seq reads from four representative samples were used to generate a reference transcriptome assembly to support downstream gene expression analysis, following Honaas et al. (2016). The RNA transcripts were assembled on the Washington State University’s High Performance Computing (HPC) system using Trinity v2.11.0, where the minimal kmer coverage was set to ‘3’ and the flags ‘--trimmomatic’ and ‘--no_parallel_norm_stats’ were used to filter the reads for quality and skip the high-mem normalization stats generator step, respectively. The assembled transcripts were further processed using PlantTribes2 tool AssemblyPostProcessor with the implemented Transdecoder option, where transcripts shorter than 300 base pairs (bp) were removed. A summary of the assembly result was generated with the TrinityStats.pl script provided in the Trinity package. In addition, a BUSCO v5.0.0 analysis was performed with the eudicot_odb10 database to evaluate assembly completeness.

### 2.5 Candidate gene selection

To select maturity-related genes for qPCR analysis, we cross-referenced the recently published RNA-seq studies on pear maturity (Honaas et al., 2021). The reference genomes used in that study differ in assembly and annotation quality. Furthermore, no exact gene-to-gene comparison exists between the two genomes, making it impossible to identify shared genes between the two studies. To circumvent this knowledge gap, we instead searched for shared orthogroups. First, genes associated with different maturity levels identified in the two studies were classified into precomputed PlantTribes2 26Gv2.0 orthogroups using both BLAST and HMM classifiers, as implemented in the *GeneFamilyClassifier* tool. A total of 141 orthogroups shared by the two studies were identified (**Supplementary Table 2**). To narrow down the list of candidate genes, we first excluded large orthogroups in *Pyrus. communis*. For the remaining orthogroups, the transcripts from the *de novo* assembly and 13 other Rosaceae species or cultivars (*Rosa chinensis* ‘Old Blush’ (Raymond et al., 2018), *Rubus occidentalis* v3 (VanBuren et al., 2018), *Fragaria vesca* v2.0a2 (Li et al., 2018), *Prunus avium* (Shirasawa et al., 2017), *Pyrus communis* ‘d’Anjou’ (Zhang et al., 2022), ‘Bartlett’ v1 (Chagné et al., 2014), ‘Bartlett double haploid v2’ (Linsmith et al., 2019), *Pyrus bretschneideri* (Xue et al., 2018), *Malus domestica* ‘Hanfu’ (Zhang et al., 2019), ‘Golden Delicious double haploid 13’ (Daccord et al., 2017), ‘Gala’, *Malus sieversii*, and *Malus sylvestris* (Sun et al., 2020). PlantTribes2 26Gv2.0 classification of those genomes were obtained from Zhang et al. (2022), and sequences classified into the same orthogroup were integrated with the scaffold sequences. Orthogroup multiple sequence alignment and phylogeny were processed on SCINet (https://scinet.usda.gov/) using PlantTribes2’s *GeneFamilyAligner* and *GeneFamilyPhylogenyBuilder* tools with MAFFT and fasttree, respectively. Next, the orthogroup alignments and phylogenies were manually examined and additional filtering steps were applied to further narrow down the candidate list: 1) orthogroups with bad candidate gene models (i.e. fragmented or low homology sequences) were excluded; 2) orthogroups with previously reported maturity-related functions were prioritized. After filtering, 10 orthogroups (**Supplementary Table 2**) highlighted with a yellow background in **Supplementary Table 2** were selected for further analysis. Ortholog of the maturity related candidate genes were determined based on the gene family phylogeny (**Supplementary File A**).

### 2.6 Primer development and qPCR

Primers were developed in Primer3Web v4.1.0 (https://primer3.ut.ee/) with “*Product Size Ranges*” set to 70-150bp and “*Number To Return*” to 20. For candidate genes with highly similar homologous sequences, primer development targeted regions showing the greatest dissimilarity. Depending on the number of orthologous transcripts identified, one to three sets of primers were designed for each orthogroup of interest. We first tested 19 primer pairs developed across 10 orthogroups to determine which primer pairs performed the ‘best’ (good melt curves, low variation across biological replicates, detectable differences in relative expression) for each orthogroup of interest on a subset of samples (pre- and at-harvest samples). Based on the initial qPCR results, we selected the ‘best’ performing primer pair for each OG of interest and performed qPCR on the postharvest samples. We first excluded candidate genes that showed no expression differences across samples. If multiple candidate genes show the same expression pattern across samples, we kept only the one with the highest expression level. Candidate genes and their primer characteristics can be found in **Supplementary Table 1** (transcript sequences, primer alignments, and regions excluded for primer design are detailed in **Supplementary File B**). Two reference genes, PCP007544 from Honaas et al. (2021) and PCP030439 from Busatto et al. (2021) were selected.

Primers were synthesized by Integrated DNA Technologies (IDT, Coralville, IA), dissolved in qPCR-grade water (catalog no. W4502; Sigma-Aldrich, St. Louis, MO) to produce 100 µm solutions, and stored at –20 °C. qPCR was performed as described in Hargarten et al. (2018) for ‘Granny Smith’ on all samples with slight modifications made for automated liquid handling with an epMotion 5073 (Eppendorf, Hamburg, Germany) as described in Waite et al. (2023). All qPCR assays included reverse transcription-negative controls and no-template controls. Each reaction was run in technical triplicate. The “sample maximization” scheme described by Hellemans et al. (2007) was followed, in which all samples in each sample set were run on each plate, obviating the need for inter-run calibrators. Amplification and melt curves for each triplicated group of technical replicates were manually inspected for Ct variance and melt curve anomalies. Reaction efficiency for each GOI was calculated from the raw Quantification Amplification Results (QAR) downloaded from sample Set 1 plate optical files generated by Bio-Rad’s CFX Maestro Software 1.0 (4.0.2325.0418) using the R v4.1.2 (R Core Team, 2021) package ‘qpcR’ v1.4-1 68 (Ritz and Spiess, 2008; Spiess, 2018), following methods described in Waite et al. (2023).

### 2.7 Metabolome analysis by LC-MS and data processing

The non-polar extraction was conducted following the detailed protocol described by Rudell et al. (2009); Leisso et al. (2016), and Yoo et al. (2023) using frozen peel powder (0.5 g). For analysis, 0.5 µL of the extract was injected into an Agilent 1100 HPLC system equipped with a Chromolith Performance RP-18e (4.6 × 100 mm) monolithic column (EMD Millipore Corporation). A linear gradient elution was applied using of methanol/water (80:20, v/v) as Solvent A and ethyl acetate as Solvent B. The flow rate was maintained at 1 mL min^-1^, and the column temperature maintained at 20 °C. The elution program held Solvent A for the initial 2 minutes, gradually increased Solvent B to 65% B by 21 minutes and maintained this mixture until 35 minutes. Mass spectrometric detection was performed using an Agilent 6230 time-of-flight mass spectrometer equipped with an atmospheric pressure chemical ionization (APCI) source. Both positive and negative mass spectra were acquired in fast polarity switching (FPS) mode across a range of 100–1700 m/z. Key APCI source settings included: drying gas (N_2_) temperature at 350 °C, drying gas flow at 4 L min^-1^, nebulizer pressure at 60 psig, vaporizer temperature at 400 °C, corona current at 4 µA (positive mode), capillary voltage at 3000 V (positive mode), and fragment or voltage at 225 V (positive mode).

Instrument control and data acquisition were carried out using the Agilent MassHunter Quantitative software (version 10.1). Mass spectral tags (MSTs) for metabolites identified either confirmed with authentic standards or tentatively assigned based on spectral interpretation and/or similarity to compounds of the same class are listed in **Supplementary Table 3**. The deconvolution of mass spectra and assignment of MSTs to the in-house reference library followed protocols described by McTavish et al. (2025); Rudell et al. (2017, 2009), and (Sánchez-Contreras et al., 2021).

### 2.8 Statistical analysis

Data were analyzed by analysis of variance (ANOVA) to evaluate the effects of orchard, storage treatment, storage duration, and their interactions. Mean comparisons were performed using Tukey’s honestly significant difference (HSD) test at P ≤ 0.05. Overall metabolite composition was assessed by permutational multivariate analysis of variance (PERMANOVA) using samples collected after 30 and 60 d of storage, when all storage treatments (RA, SWC, and CA) were represented. Principal component analysis (PCA) was performed independently for each storage duration using normalized abundance data for all 311 identified metabolites. To identify significantly affected genes and metabolites, ANOVA models were fitted in R (version 4.4.3), and P-values were adjusted using the Benjamini–Hochberg false discovery rate (FDR). Variables with FDR-adjusted P < 0.05 were considered significant. Associations between gene expression and metabolite abundance were evaluated using Spearman correlation analysis with Benjamini–Hochberg FDR correction, and only significant correlations (FDR-adjusted P < 0.05) were retained. Graphical visualizations were generated using SigmaPlot 10.0, R (version 4.4.3), and MetaboAnalyst 6.0.

## 3 Results

### 3.1 Effect of CA and SWC on fruit maturity and superficial scald incidence in the orchard

The physiological maturity of ‘Packham’s Triumph’ pears at harvest and during storage showed significant differences among the three orchards (**Table 1**). At harvest, Orchard 2 exhibited the highest firmness (72.9 N) and starch index (2.3), while Orchards 1 and 3 showed lower starch indices of 1.48 and 1.55 (data not shown), respectively. During storage, different patterns of fruit physiological quality were observed among orchards and treatments (**Table 1**). In Orchards 1 and 2, fruit stored under RA conditions maintained significantly higher firmness (66.5 N and 80.5 N at 60 days, respectively) compared to SWC and CA stored fruits. Conversely, in Orchard 3, the CA treatment was more effective at maintaining firmness (69.5 N) than the SWC treatment (60.7 N) at 60 d. SSC values also varied by storage atmosphere. In Orchard 1, SWC and CA treatments resulted in higher SSC (14.5–15.1°Brix) compared to RA (12.8°Brix) after 60 d. In Orchard 3, the highest SSC was observed in the SWC (15.9°Brix). The *I*_AD_ values remained relatively high across most treatments, though Orchard 3 showed a notably lower value in the CA group at 30 d (1.5) compared to RA (1.7) and SWC (1.9).

**Table 1:** Fruit maturity (firmness, soluble solids, *I*_AD_) at harvest, 30, 60 d (days) and SS incidence (%) after 140 and 180 D in ‘Packham’s Triumph’ pears from different orchards, stored in under regular atmosphere (RA, -0.5 °C and >90%RH), stepwise cooling (SWC; 1 week at 5 °C, 1 week at 3 °C, 1 week at 2 °C and the rest at -0.5 °C and >90%RH), and controlled atmosphere (CA, O_2_: 2.0 kPa, CO_2_: 1.0 kPa, -0.5 °C and >90%RH) plus 14 D shelf life, 20 °C from three orchards.

Orchard (O)	Treatment (T)	Firmness (N)			Soluble Solids (Brix)			*I*_AD_ (0-2.2)			SS (%)	
		Harvest	30 d	60 d	Harvest	30 d	60 d	Harvest	30 d	60 d	140 + 14d	180 + 14d
# 1	RA	69.6 a^z^	68.9 b	66.5 b	13.5 b	12.0 a	12.8 a	1.84 a	1.4 a	1.8 ab	58.0 c	79.0 c
	SWC		61.8 a	61.9 a		14.6 b	14.5 b		1.8 b	1.7 b	52.0 b	46.0 b
	CA		64.7 ab	62.3 a		15.2 b	15.1 b		1.8 b	1.9 a	0.0 a	2.0 a
# 2	RA	72.9 b	74.3 b	80.5 b	12.9 ab	14.3	13.2	1.86 a	1.8	2.0	92.0 c	100.0 b
	SWC		62.4 a	72.1 a		14.9	13.4		1.9	2.0	90.0 b	100.0 b
	CA		73.1 b	70.3 a		14.4	13.6		1.8	2.0	74.0 a	85.0 a
# 3	RA	72.5 b	68.1 b	62.9 a	12.2 b	13.8 a	14.8 b	1.92 b	1.7 b	1.9	61.0 c	75.0 c
	SWC		59.6 a	60.7 a		15.4 b	15.9 b		1.9 c	1.8	42.0 b	30.0 b
	CA		68.9 b	69.5 b		13.4 a	11.7 a		1.5 a	1.8	0.0 a	4.0 a
Orchard	*P* value		***	***		**	**		***	***	***	***
O x T	*P* value		*	***		***	***		***	**	*	***

^Z^ Different letters indicate significant differences between treatments (Tukey, *P ≤* 0.05). Orchard× Treatment interaction: NS, *, **, or *** = not significant or significant at p < 0.05, 0.01, or 0.001.

Superficial scald (SS) incidence was strongly influenced by both orchard and storage conditions, with a significant orchard × treatment interaction (**Table 1**). CA storage effectively suppressed SS development, maintaining rates between 0% and 4% in Orchards 1 and 3. In contrast, Orchard 2 remained highly susceptible, with SS incidence of 74–85% even under CA conditions. RA storage consistently resulted in the highest SS incidence, ranging from 58% to 100% across orchards. SWC partially mitigated SS in Orchards 1 and 3, reducing incidence to 30–52% compared with RA, but showed minimal to no protective effect in Orchard 2, where scald incidence remained extremely high (90–100%). Overall, Orchard 2 fruit consistently exhibited the highest susceptibility to SS across storage treatments, indicating strong orchard-dependent variation in susceptibility to the disorder.

### 3.2 Reference transcriptome assembly and quality assessment

A total of 139 million bases were assembled, resulting in 44,751 genes and 117,239 transcripts with contig N50s of 1664 bp and 1767 bp, respectively. The number of genes was reduced to 21,904, and the number of transcripts was reduced to 77,448 post-processing with the *AssemblyPostProcessor* tool from PlantTibes2. The contig N50s of genes and transcripts were reduced to 1473 bp and 1440 bp, respectively. The complete record of transcripts was reduced to 1473 bp and 1440 bp, respectively. The complete record of transcript statistics can be found in the **Supplementary File C-1**. The post-processed transcripts were searched against the eudicot_odb10 database, and 2047 (88.0%) complete Benchmarking Universal Single Copy Orthologs (BUSCOs) were identified (complete BUSCO output can be found in **Supplementary File C-2**), which is higher than the annotation BUSCO from Bartlett. DH_v2 genome (81.8%) and only slightly lower than the other two European pear genome annotations (92.9-93.7%) (Zhang et al., 2022). Since the tissues used for transcriptome assembly were collected only from the fruit, it is expected to include fewer BUSCO genes than whole-genome annotations. The fact that the *de novo* assembly’s complete BUSCO score is close to whole-genome annotation indicates that the assembly captured most genes at the expected length, thus a high-quality assembly. This assembly was used as a reference for quantifying expression of selected genes and was not intended as a comprehensive transcriptome-wide analysis.

### 3.3 qPCR candidate selection

A detailed workflow for selecting candidate genes from RNA-seq-derived orthogroups, including filtering, phylogenetic validation, and qPCR screening, is provided in **Supplementary Figure 1**. Several recent studies have examined genes associated with fruit maturity or superficial scald, a maturity-related disorder, using both metabolomic and transcriptomic approaches (Lindo-García et al., 2020; Busatto et al., 2021; Honaas et al., 2021). We used qPCR to test if those maturity-linked genes also showed different expression patterns in our samples. To narrow down the thousands of genes identified from the two recent transcriptome experiments, we cross-reference the candidate genes. However, the reference genomes used in these two studies differed in assembly and annotation quality. Furthermore, no exact gene comparison exists between the two genomes, making the identification of shared genes between the two studies impossible. To circumvent this knowledge gap, we instead searched for shared orthogroups. A total of 141 orthogroups were shared between the two studies, and we further narrowed the list by excluding large orthogroups and poor-quality gene models, and by investigating orthogroup function (**Supplementary File A**). A final set of 10 orthogroups with 19 candidate genes was selected initially for qPCR analysis (**Supplementary Table 2**).

The candidate genes were selected from the ‘Bartlett’ genome annotations, and, due to domestication and hybridization in pears, the level of polymorphism between pear cultivars is expected to be high. To ensure that our primer will amplify the target genes in ‘Packham’s Triumph’, we identified the orthologs of the literature candidate genes from the *de novo* assembly using tools from PlantTribes2, and designed primers using the ‘Packham’s Triumph’ *de novo* transcripts.

Initial qPCR results using pre- and at-harvest samples showed that, for most candidate genes, differences in expression were observed among fruits from different locations (**Supplementary Figure 2**). Additionally, genes exhibit similar expression patterns across samples (**Supplementary Figure 3**). To expand our gene expression exploration to more samples, we further reduced the number of candidate genes to one gene per orthogroup by removing those with no significant differences in expression levels across samples and those that share an expression pattern with other genes in the same orthogroup but have a lower relative expression level.

### 3.4 Gene expression response over time, storage condition, and orchard

**Figure 1:**
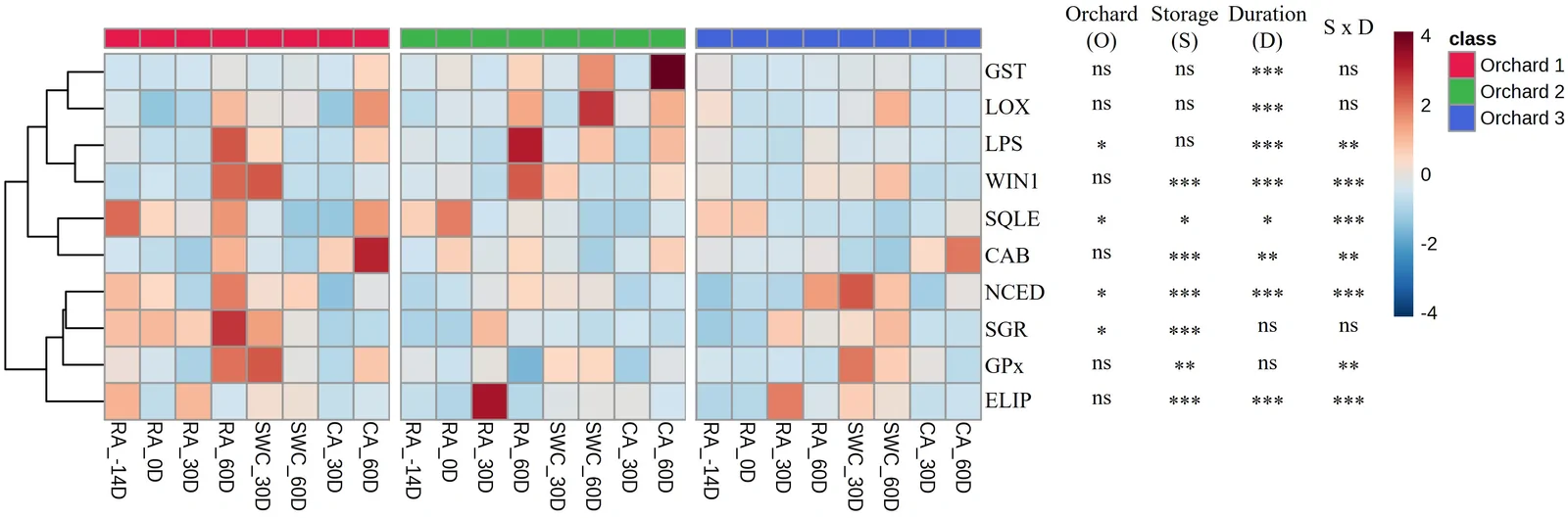
Heatmap of relative expression levels (qPCR) of 10 target genes in the peel tissue of ‘Packham’s Triumph’ pears sampled 14 days before harvest (-14 D), at harvest (0 D), and after storage for 30 (30D) and 60 (60D) days under regular atmosphere (RA, -0.5 °C and >90%RH), stepwise cooling (SWC; 1 week at 5 °C, 1 week at 3 °C, 1 week at 2 °C and the rest at -0.5 °C and >90%RH), and controlled atmosphere (CA, O_2_: 2.0 kPa, CO_2_: 1.0 kPa, -0.5 °C and >90%RH) from three orchards. Each data point indicates the mean of four replicates (n = 4) with five fruits per replicate. NS, *, **, or *** = not significant or significant at p < 0.05, 0.01, or 0.001, respectively; indicate significant differences based on ANOVA followed by Tukey’s HSD. Genes shown include *Chlorophyll A/B binding protein* (CAB), *Senescence-inducible chloroplast stay-green protein* (SGR), *Ethylene-responsive transcription factor WIN1* (WIN1), *Early light-induced protein* (ELIP), *9-cis-epoxycarotenoid dioxygenase* (NCED), *Squalene epoxidase/Squalene monooxygenase* (SQLE), *Lipase* (LPS), *Glutathione peroxidase* (GPx), *Lipoxygenase* (LOX), *Glutathione S-transferase* (GST).

Transcript abundance of 10 genes associated with oxidative stress, senescence, and defense responses was assessed by qPCR. Distinct transcriptional profiles were observed among Orchard 1, Orchard 2, and Orchard 3 in response to storage atmosphere (RA, CA, and SWC) and storage duration (-14, 0, 30, and 60 d), as illustrated in the heatmap (**Figure 1**) and supplementary figure (**Supplementary Figure 3**). Analysis of variance revealed that storage duration significantly affected the expression of all genes except stay-green protein (SGR) and glutathione peroxidase (GPx) (**Figure 1**). Orchard significantly influenced lipase (LPS), squalene epoxidase (SQLE), 9-cis-epoxycarotenoid dioxygenase (NCED), stay-green protein (SGR), whereas storage atmosphere significantly affected SQLE, ethylene-responsive transcription factor WIN1, chlorophyll a/b-binding protein (CAB), NCED, SGR, glutathione peroxidase (GPx), and early light-induced protein (ELIP). Significant storage-by-duration interactions were detected for all genes except GST, lipoxygenase (LOX), and SGR.

Overall, antioxidant- and stress-related genes showed distinct treatment-dependent expression patterns (**Figure 1**). GST was strongly induced under CA after 60 d in Orchard 2, whereas LOX accumulated primarily after prolonged storage under CA in Orchard 1 and SWC in Orchard 2. LPS expression was highest under RA after 60 d in Orchards 1 and 2. Among ripening and senescence-related genes, WIN1, CAB, NCED, and SGR generally exhibited greater transcript abundance during storage, particularly under RA or CA, depending on the orchard, while SQLE showed relatively high expression before storage and at early storage stages. GPx and ELIP displayed orchard-dependent responses, with elevated expression under RA or SWC during later storage. These results demonstrate that storage atmosphere and duration regulated stress-and senescence-related gene expression in an orchard-dependent manner.

### 3.5 Storage- and orchard-specific metabolite profiles

**Figure 2:**
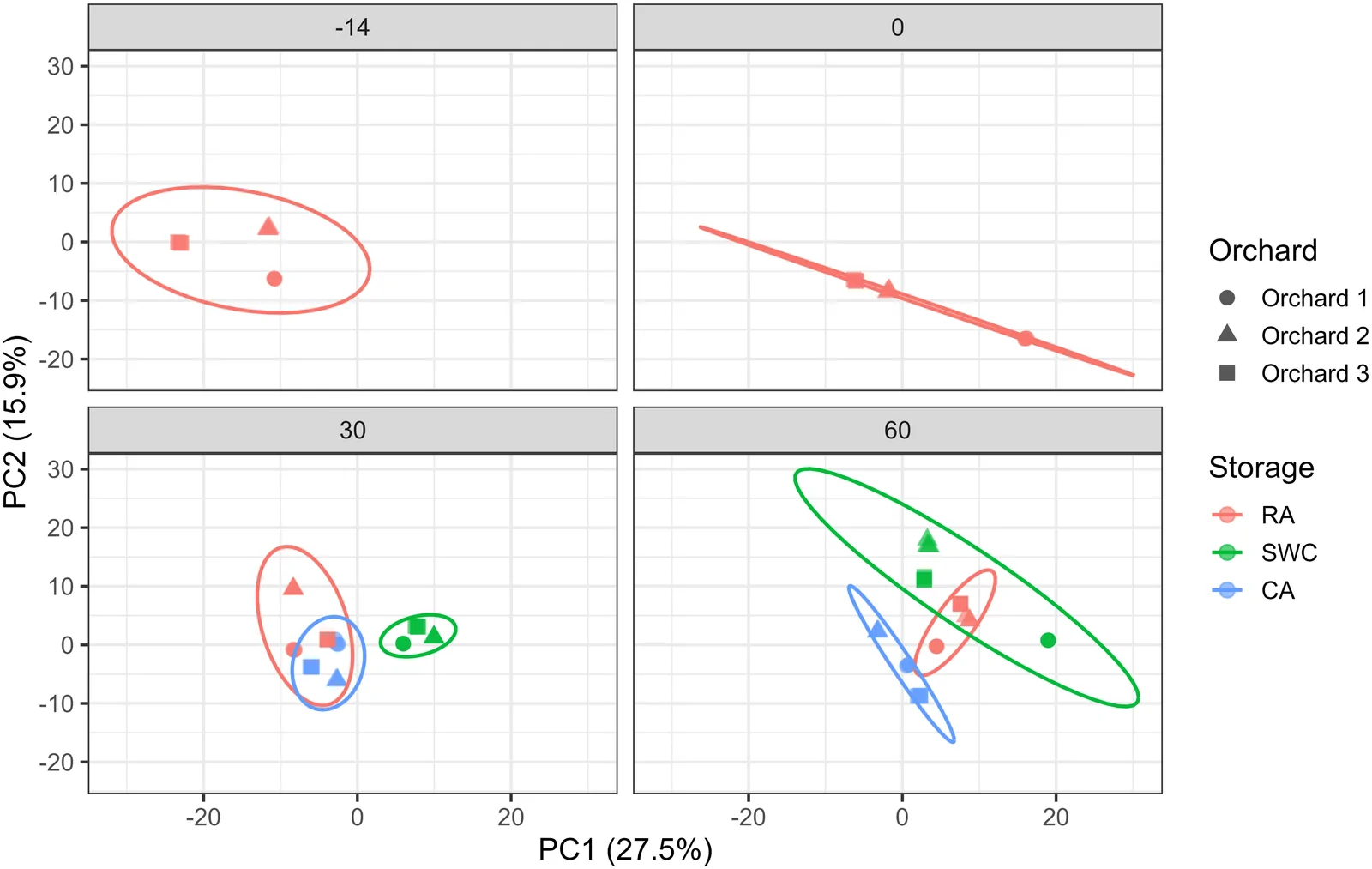
Principal component analysis (PCA) of metabolite profiles across orchards, storage treatments, and storage durations at 60 days. PCA was performed using log-transformed and scaled metabolite abundance data. colored by storage treatment (RA, regular atmosphere; SWC, stepwise cooling; CA, controlled atmosphere) and shaped by orchard (Orchard 1, ●; Orchard 2, ▲ Orchard 3, ■);. Panels represent individual storage durations. PC1 and PC2 explain 26.9% and 15.4% of the total variance, respectively.

To evaluate the contribution of orchard effects to overall metabolite variation, a principal component analysis (PCA) was performed using all samples across orchards, storage treatments, and storage durations (**Figure 2**). PCA indicated modest orchard-related variation before storage, particularly at −14 d, suggesting differences in fruit physiological status prior to storage. At 0 days, orchards showed minimal separation, but distinct treatment effects emerged after 30 and 60 days. At 30 d, SWC samples formed a separate cluster from RA and CA fruit, while orchard-related differences remained evident within the RA and CA groups. At 60 d, SWC samples were clearly separated from RA and CA samples along PC1, whereas CA samples were also distinguished from RA fruit, indicating increasingly divergent metabolic responses among storage treatments over time. PERMANOVA performed using the 30- and 60-d samples, where all storage treatments were represented, demonstrated significant effects of orchard (R² = 0.085, P < 0.0001) and the Storage × Duration interaction (R² = 0.144, P < 0.0001) on metabolite composition. These results indicate that both preharvest orchard factors and storage-dependent temporal responses contributed significantly to metabolic variation.

**Figure 3:**
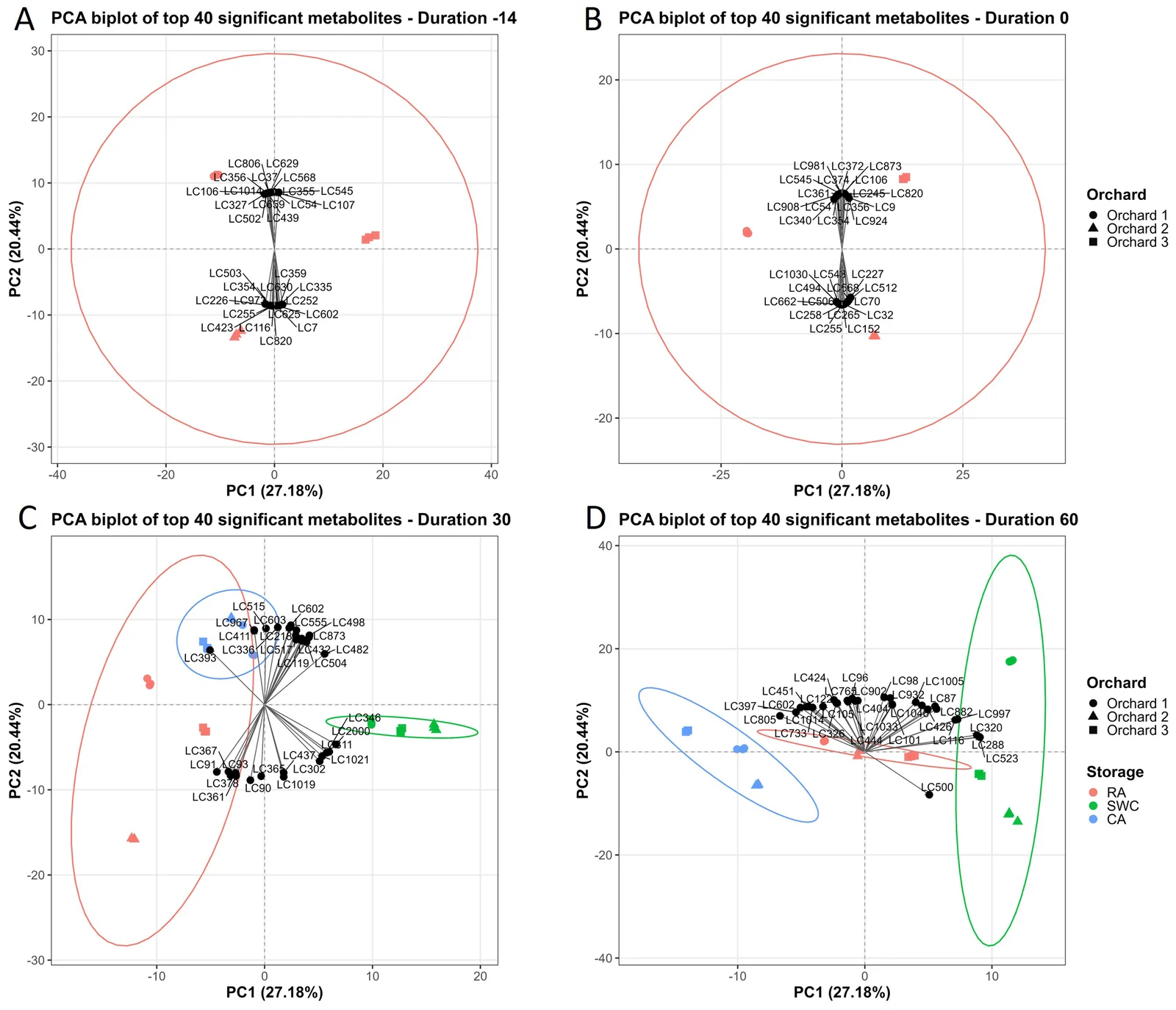
Principal component analysis (PCA) biplots of the top 30 FDR-significant metabolites at each storage duration: **(A)** Duration−14, **(B)** Duration 0, **(C)** Duration 30, and **(D)** Duration 60. PCA was performed independently for each storage duration using all detected metabolites. Points represent individual fruit samples, colored by storage treatment (RA, regular atmosphere; SWC, stepwise cooling; CA, controlled atmosphere) and shaped by orchard (Orchard 1, ●; Orchard 2, ▲ Orchard 3, ■);. Arrows indicate metabolite loading vectors, and only the 30 metabolites with the highest loading strengths are labeled for clarity. The direction of each loading vector indicates the association of the metabolite with sample distribution, while the vector length reflects its relative contribution to the separation along PC1 and PC2. The percentages shown on the axes indicate the proportion of total variance explained by each principal component for the corresponding storage duration.

To identify the metabolites contributing to sample separation, PCA biplots were generated independently for each storage duration (-14, 0, 30, 60 d) using the normalized abundance matrix of all 311 identified metabolites. For clarity, only the 30 metabolites with the highest absolute loadings on PC1 and PC2 were labeled in each biplot using their LC codes (**Figure 3**), which correspond to the metabolite annotations in **Supplementary Table 3**. At -14 d and 0 d, samples showed limited separation among treatments, indicating relatively similar metabolite profiles before and at the beginning of storage (**Figures 3A, B**). At these early stages, separation was mainly associated with sterol esters and lipid-related metabolites, including campesteryl linolenate (LC107), campesteryl linoleate (LC106), campesterol (LC545), β-sitosteryl glucosyl stearate (LC423), diacylglycerols (DAGs; LC354, LC355, LC356, LC340), violaxanthin (LC503), and unknown pentacyclic triterpenols (LC116, LC502, LC806).

By 30d, clearer treatment separation was observed, particularly between CA and SWC samples (**Figure 3C**). This separation was associated with changes in phytosterols and lipid related compounds, including β-sitosterol (LC336), stigmasteryl glucosyl palmitate (LC967), β-sitosteryl glucosyl palmitate (LC411), sphingolipids (LC90, LC91, LC93, LC361, LC367, LC378), triglycerides (TAGs; LC119, LC437), monogalactosyldiacylglycerols (MGDGs; LC302), uvaol (LC498), pomaceic acid (LC2000), and unknown pentacyclic triterpenols (LC346, LC482, LC504, LC515, LC517).

After 60 d, treatment-dependent separation was strongest, with SWC clearly separated from RA and CA along PC1 and PC2 (**Figure 3D**). Metabolites contributing to this separation included triterpenoid and phytosterol conjugates such as uvaol palmitate (LC98), uvaol stearate (LC101), erythrodiol palmitate (LC96), erythrodiol linoleate (LC87, LC932), α-amyrin linoleate (LC451), α-amyrin linolenate (LC444), β-amyrin (LC326), cycloartenyl linoleate (LC122), stigmasteryl palmitate (LC1046), and hydroxycinnamoyl acyl esters, including p-coumaryl stearate (LC288), p-coumaryl oleate (LC523), and p-coumaryl eicosanoate (LC320). Overall, PCA indicated that storage treatment effects on metabolite composition became more pronounced as storage duration increased, with the greatest metabolic divergence observed after 60 d (**Figure 3D**).

### 3.6 Storage-associated metabolites and coordinated stress responses

**Figure 4:**
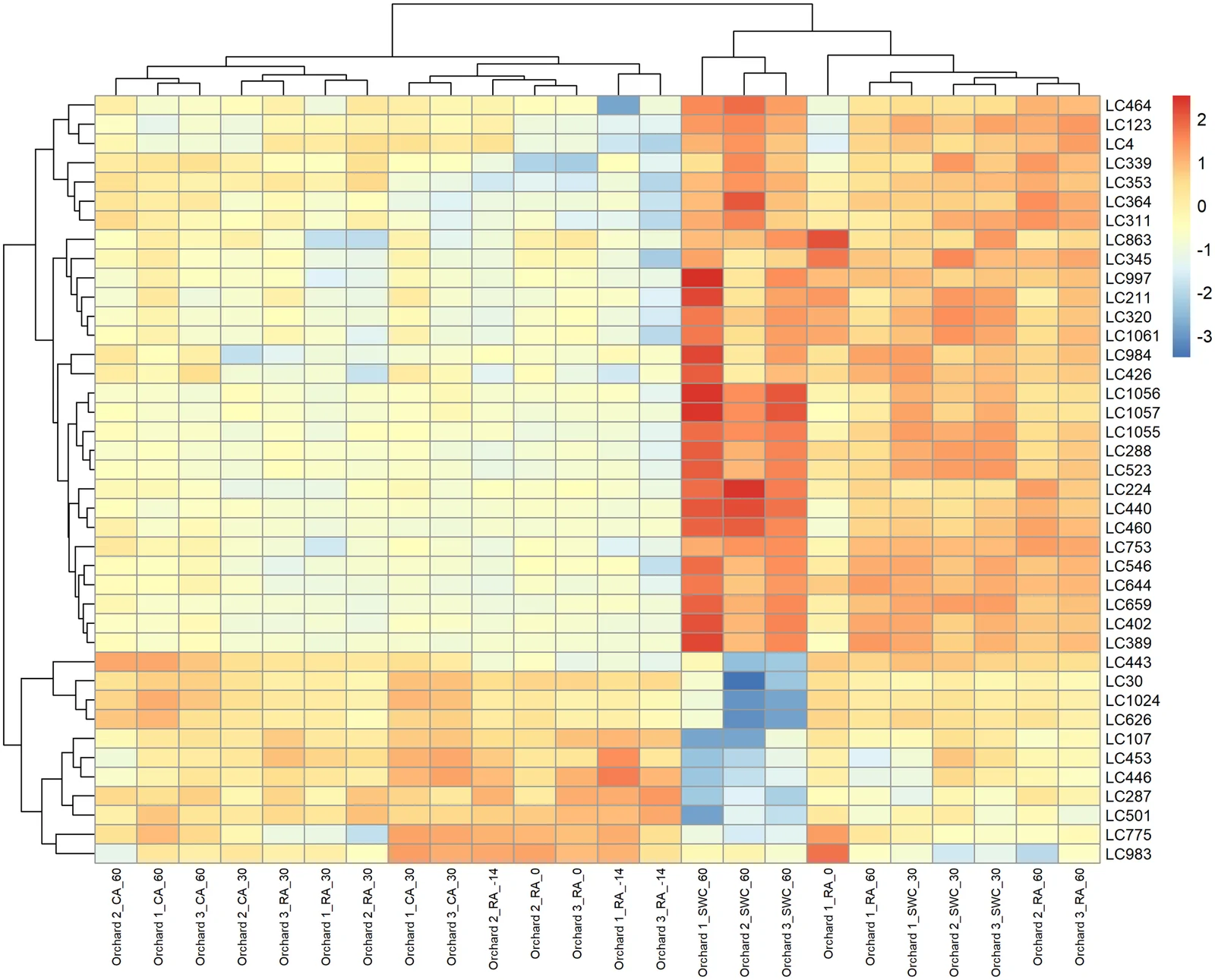
Heatmap of the top 40 FDR-significant metabolites affected by storage treatment, storage duration, or their interaction. Values represent log-transformed and row-scaled relative abundance. Variables were selected from ANOVA models including Orchard, Storage, Duration, and Storage × Duration effects, followed by Benjamini–Hochberg false discovery rate correction (FDR < 0.05).

To identify variables significantly associated with storage treatment, storage duration, and their interaction, ANOVA models including the effects of Orchard, Storage, Duration, and Storage × Duration were fitted, followed by Benjamini–Hochberg false discovery rate (FDR) correction. Hierarchical clustering of the top 40 FDR-significant variables revealed that storage treatment was the primary factor of metabolic variation (**Figure 4**). A distinct cluster of metabolites accumulated predominantly under SWC and CA storage at 60 d, including several hydroxycinnamoyl acyl esters, such as LC523 (p-coumaryl oleate), LC659 (p-coumaryl linolenate), and LC288 (p-coumaryl stearate), as well as triterpene-related compounds, including LC183 (farnesyl linoleate), LC644 (farnesyl linolenate), and LC389 (farnesyl oleate). Several unidentified metabolites, including LC1055, LC1056, and LC1057, also showed strong accumulation under SWC and CA conditions.

In contrast, metabolites with lower abundance under SWC at 60 d included phytosterol conjugates LC453 (β-sitosteryl linoleate), LC446 (β-sitosteryl linolenate), and LC107 (campesteryl linolenate), as well as TAG-related metabolites LC30 and LC626. Several diacylglycerols (LC353, LC364) and sterol conjugates were primarily associated with storage duration, whereas LC443 and LC626 exhibited significant interactions between storage and duration, indicating treatment-specific temporal responses. Overall, these findings demonstrate that prolonged storage under SWC and CA promoted the accumulation of hydroxycinnamoyl esters and terpenoid-related metabolites while altering sterol and membrane lipid metabolism (**Figure 4**).

**Figure 5:**
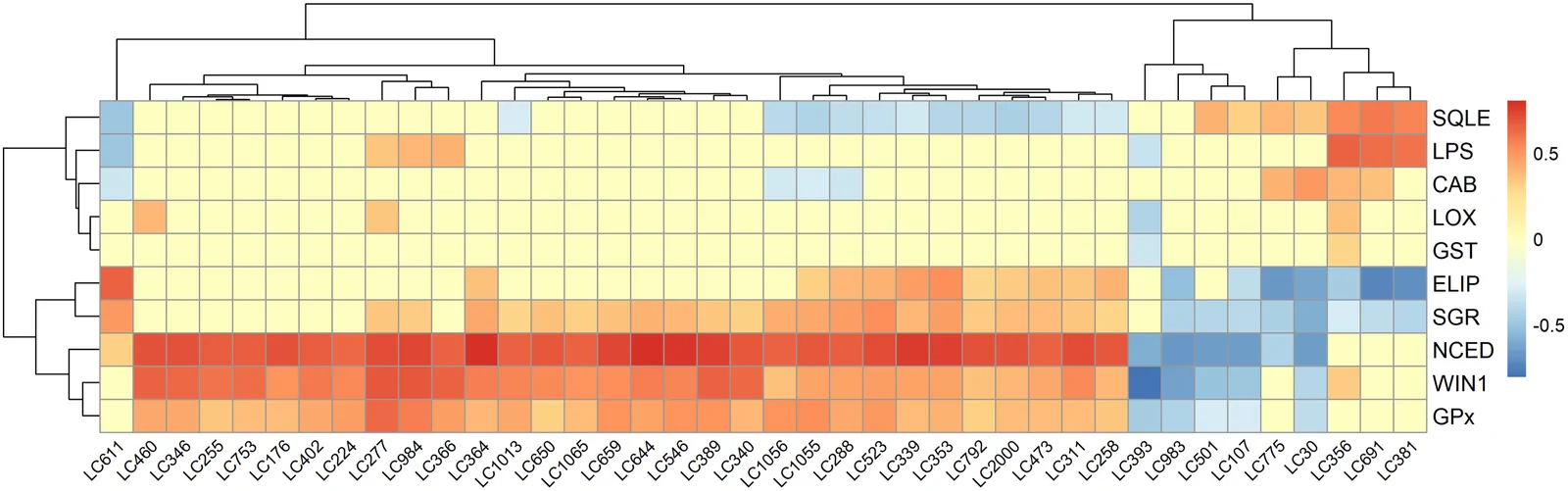
Heatmap of the top FDR-significant Spearman gene–metabolite correlations. Correlations were calculated between gene expression and metabolite abundance across all samples. P-values were adjusted using the Benjamini–Hochberg false discovery rate procedure. Only correlations with FDR-adjusted P < 0.05 are shown.

To investigate coordinated transcriptional and metabolic responses, FDR-significant Spearman correlations were calculated between gene expression and metabolite abundance (**Figure 5**). NCED, WIN1, and GPx exhibited the most extensive positive correlation networks, followed by SGR, whereas ELIP displayed predominantly negative correlations with many of the same metabolites. In contrast, CAB, GST, and LOX showed relatively few significant associations, suggesting more limited relationships with metabolite accumulation. Among the evaluated genes, NCED exhibited the strongest correlation network, showing positive associations with membrane lipid metabolites, including diacylglycerols (LC353 and LC364), farnesyl conjugates (LC546, LC644, and LC389), hydroxycinnamoyl acyl esters (LC288 and LC523), and several trienol-related metabolites (LC176, LC224, LC440, and LC460). In contrast, NCED was negatively correlated with campesteryl linolenate (LC107), neoxanthin (LC501), triacylglycerol (LC30), and β-sitosteryl glucosyl conjugates (LC393 and LC983). Similar positive associations with diacylglycerols, farnesyl conjugates and hydroxycinnamoyl acyl esters were observed for WIN1 and GPx. Likewise, SGR was positively correlated with p-coumaryl stearate (LC288), p-coumaryl oleate (LC523), diacylglycerols (LC353 and LC364), and sphingolipid LC611. In contrast, ELIP exhibited negative correlations with several lipid-related metabolites, including diacylglycerol LC381, triacylglycerol LC30, β-sitosteryl glucosyl oleate (LC983), and two unidentified metabolites (LC691 and LC775), while maintaining positive associations with LC353 and LC523. Collectively, these results indicate coordinated regulation of stress-responsive genes with metabolites involved in membrane lipid remodeling, terpenoid metabolism, and hydroxycinnamoyl ester biosynthesis during storage.

## 4 Discussion

### 4.1 Orchard-dependent variation strongly influences superficial scald susceptibility

Superficial scald is a major postharvest disorder of apples and pears that has been closely associated with fruit maturity, oxidative stress, membrane integrity, and pre- and postharvest environmental conditions (Lurie and Watkins, 2012; Whitaker, 2013; Marc et al., 2020; Fernández-Cancelo et al., 2022). In the present study, orchard origin was a major determinant of superficial scald susceptibility, with Orchard 2 consistently exhibiting substantially higher disorder incidence than Orchards 1 and 3 regardless of storage atmosphere (**Table 1**). Although controlled atmosphere (CA) storage effectively suppressed superficial scald in Orchards 1 and 3, its effectiveness was limited in Orchard 2, indicating that postharvest storage alone could not fully overcome the intrinsic susceptibility established before harvest. These findings are consistent with previous studies in ‘Packham’s Triumph’ and ‘Abate Fetel’ pears, which demonstrated that orchard-specific physiological status strongly influences superficial scald development and the efficacy of postharvest storage strategies (Bonora et al., 2021; Torres et al., 2021).

Previous studies have shown that fruit susceptibility to superficial scald is largely determined by preharvest physiological status, including fruit maturity, oxidative balance, ethylene metabolism, and antioxidant capacity (Calvo et al., 2015; Lindo-García et al., 2020; Torres et al., 2021). In addition, Caracciolo et al. (2020) reported that differences in antioxidant systems during cold storage were closely associated with superficial scald development in pear, further supporting the importance of the fruit’s physiological condition at harvest. Likewise, Dällenbach et al. (2020) demonstrated that both pre- and postharvest factors contribute to superficial scald incidence in apple, emphasizing that storage treatments cannot completely compensate for differences established during fruit development. Our results agree with these findings, demonstrating that orchard origin significantly influenced both superficial scald susceptibility and subsequent metabolic responses (**Figure 2**).

Metabolomic analysis further supported the importance of orchard effects. Orchard separation was relatively limited before storage but became increasingly evident after 30 and 60 d as fruit responded differently to the storage atmospheres (**Figures 2**, **3**). This pattern suggests that orchard origin primarily influenced the capacity of fruit to adapt metabolically during prolonged cold storage rather than determining the initial metabolite profile. Similar storage-dependent metabolic reprogramming has been reported in apple, where progressive metabolic shifts precede superficial scald development and reflect differences in fruit physiological status and stress adaptation (Rudell et al., 2009; Busatto et al., 2018; Populin et al., 2023).

Collectively, these findings demonstrate that orchard origin is a key determinant of superficial scald susceptibility by shaping the fruit’s physiological status before storage and its subsequent metabolic adaptation during cold storage. Therefore, the effectiveness of postharvest storage technologies should be considered in the context of orchard-specific fruit physiology rather than as universally applicable treatments.

### 4.2 Effects of storage strategies on superficial scald

Controlled atmosphere (CA) storage was the most effective treatment for suppressing superficial scald, whereas stepwise cooling (SWC) provided only partial protection that varied among orchards (**Table 1**). While CA consistently suppressed superficial scald in Orchards 1 and 3, SWC was ineffective in Orchard 2, indicating that the response to storage depended on the fruit’s physiological condition before storage. Similar observations have been reported in apple and pear, where CA effectively suppresses superficial scald by reducing oxidative stress, ethylene metabolism, and α-farnesene oxidation, whereas the efficacy of alternative storage strategies depends on fruit susceptibility at harvest (Larrigaudière et al., 2019; Botes et al., 2024; Papoutsis, 2024). Reduced oxygen availability under CA likely limits ethylene production and oxidative metabolism while maintaining membrane integrity during prolonged storage, thereby reducing the physiological processes associated with superficial scald (Lurie and Watkins, 2012).

The intermediate response observed under SWC suggests that gradual cooling promoted cold acclimation but could not fully overcome the high susceptibility of Orchard 2 fruit (**Table 1**). Cold acclimation has been shown to improve tolerance to prolonged low-temperature storage by maintaining membrane integrity and enhancing antioxidant capacity prior to severe chilling stress (Marc et al., 2020). Moreover, previous studies reported that improved antioxidant systems and reduced oxidative damage were closely associated with lower superficial scald development in pear (Caracciolo et al., 2020; Niu et al., 2022). These findings are consistent with the enhanced expression of stress-related genes observed under SWC and CA in the present study (**Figure 1**), suggesting that gradual cooling activates protective responses but that their effectiveness ultimately depends on the physiological status established before harvest.

These physiological responses were further supported by changes in stress-related gene expression. Storage atmosphere significantly affected the expression of WIN1, CAB, NCED, GPx, and ELIP, whereas GST and LOX exhibited orchard-dependent responses (**Figure 1**). Similar coordinated regulation of stress-responsive genes, antioxidant metabolism, and membrane protection has been reported during successful superficial scald control in both apple and pear (Giné-Bordonaba et al., 2020; Busatto et al., 2021; He et al., 2022b; Populin et al., 2023). Collectively, these findings indicate that CA provides the most consistent suppression of superficial scald, whereas the effectiveness of SWC depends on the fruit’s capacity to activate protective physiological and molecular responses during cold storage.

### 4.3 Storage-induced metabolic remodeling during superficial scald development

Metabolomic analysis demonstrated that storage duration and atmosphere progressively reshaped fruit metabolism, with the greatest metabolic divergence occurring after 60 d (**Figures 2**-**4**). SWC and CA induced distinct metabolic profiles characterized by the accumulation of farnesyl esters (e.g., farnesyl linoleate, farnesyl oleate, and farnesyl linolenate), hydroxycinnamoyl acyl esters, and coordinated changes in diacylglycerols (DAGs), triacylglycerols (TAGs), phytosterol conjugates, and sphingolipids (**Figures 3**, **4**). These results indicate that suppression of superficial scald was associated with extensive remodeling of terpenoid and membrane-associated lipid metabolism during prolonged cold storage.

Previous metabolomic studies have shown that metabolic changes precede visible superficial scald symptoms and that successful storage strategies are accompanied by coordinated alterations in terpenoid, lipid, and sterol metabolism rather than changes in individual metabolites (Rudell et al., 2009; Busatto et al., 2018; Giné-Bordonaba et al., 2020; Populin et al., 2023). In the present study, the accumulation of farnesyl esters under SWC and CA suggests substantial modification of terpenoid metabolism under storage conditions associated with reduced superficial scald. In addition, significant changes in DAGs, TAGs, and phytosterol conjugates indicate active membrane lipid remodeling during storage.

A notable finding of this study was the consistent alteration of several phytosterol conjugates, including campesteryl and β-sitosteryl derivatives, which were among the major metabolites contributing to treatment separation (**Figures 3**, **4**). Phytosterols are important structural components of plant membranes and play essential roles in maintaining membrane organization during low-temperature storage. Maintenance of membrane sterol composition has been associated with improved membrane stability and reduced chilling-related disorders in apple (Rudell et al., 2011; Leisso et al., 2015), suggesting that similar processes may contribute to superficial scald suppression in pear.

The accumulation of hydroxycinnamoyl acyl esters under SWC and CA (**Figures 3**, **4**) further suggests activation of phenylpropanoid metabolism during storage. Hydroxycinnamoyl derivatives exhibit notable antioxidant properties, which may play a crucial role in maintaining cellular redox balance during extended cold storage. This functionality has the potential to mitigate cellular damage linked to superficial scald. Similar increases have been reported in pear fruit associated with increased antioxidant capacity and reduced superficial scald development in pear (He et al., 2022a; Niu et al., 2022), while maintenance of antioxidant systems during storage contributes to improved tolerance to oxidative stress (Caracciolo et al., 2020). Furthermore, several sphingolipids contributed to the separation of treatments (**Figure 3**), indicating that membrane remodeling extended beyond glycerolipids and phytosterols. Although their involvement in superficial scald has not been established, sphingolipids have been implicated in membrane organization and stress signaling in postharvest physiological disorders (Sánchez-Contreras et al., 2021), suggesting they may contribute to the broader membrane responses observed during prolonged storage.

### 4.4 Coordinated transcriptional and metabolic responses during storage

Correlation analysis revealed coordinated associations between stress-responsive genes and metabolites involved in terpenoid, phenylpropanoid, and membrane lipid metabolism (**Figure 5**). Among the evaluated genes, NCED, WIN1, and GPx exhibited the strongest positive correlation networks, whereas ELIP was predominantly negatively associated with several membrane lipid metabolites. These findings suggest that adaptation to prolonged cold storage involved coordinated transcriptional and metabolic responses rather than independent regulation of individual pathways.

Among the evaluated genes, NCED exhibited the strongest correlation network, showing positive associations with representative farnesyl esters, hydroxycinnamoyl acyl esters, DAGs, and selected sphingolipids (**Figure 5**). The strong association between NCED and lipid-related metabolites suggests coordination between abscisic acid (ABA)-related signaling and metabolic adaptation during cold storage. ABA is a key regulator of abiotic stress responses, and increased NCED expression may reflect activation of stress-responsive signaling that facilitates metabolic adjustment under prolonged cold conditions. Similar interactions between ABA-related pathways, lipid metabolism, and stress responses have been reported in transcriptomic studies of superficial scald (Giné-Bordonaba et al., 2020; Vittani et al., 2023). Moreover, the positive associations of WIN1 and GPx with lipid- and terpenoid-related metabolites indicate coordinated regulation between stress-responsive gene expression and membrane-associated metabolism during storage, consistent with previous transcriptomic and metabolomic studies of superficial scald in apple and pear (Giné-Bordonaba et al., 2020; Busatto et al., 2021; He et al., 2022b).

Interestingly, several campesteryl and β-sitosteryl conjugates showed negative associations with NCED, whereas DAGs, farnesyl esters, and hydroxycinnamoyl acyl esters were positively associated (**Figure 5**). These contrasting relationships suggest differential regulation of sterol metabolism and membrane lipid remodeling during prolonged storage. Similar changes in phytosterol metabolism have been associated with oxidative stress responses and membrane organization during postharvest storage (Rudell et al., 2011; Leisso et al., 2015). In contrast, GST and LOX exhibited relatively limited correlation networks despite their differential expression under specific storage conditions, suggesting that these genes contribute to storage- or orchard-specific responses rather than broad metabolic regulation.

Overall, the coordinated associations between stress-responsive genes and multiple metabolite classes indicate that successful adaptation to prolonged cold storage involves integration of transcriptional regulation with membrane lipid remodeling, antioxidant metabolism, and terpenoid metabolism. These integrated molecular responses provide a mechanistic basis for the superficial scald incidence observed under CA and SWC storage.

## 5 Conclusion

**Figure 6:**
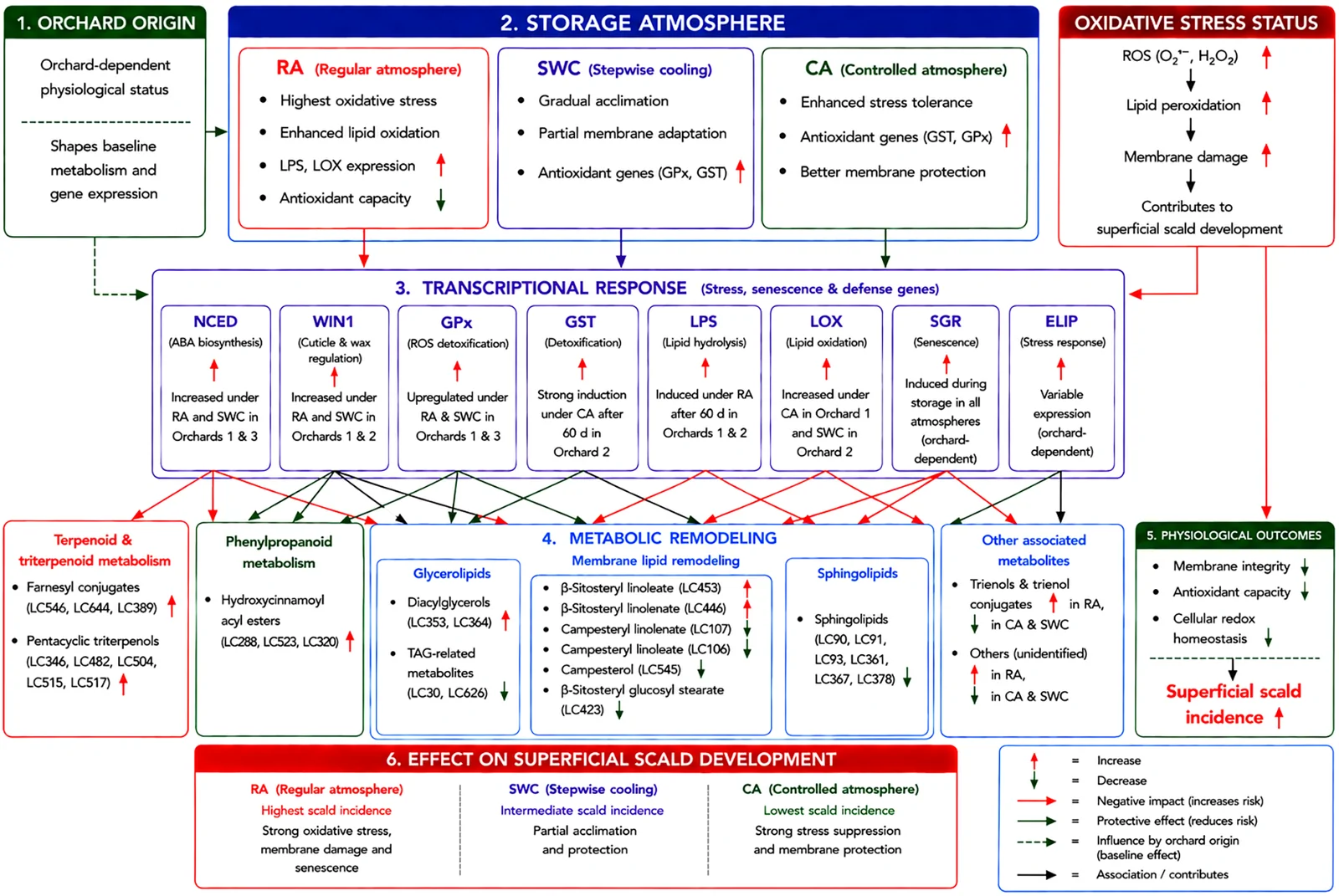
Proposed network model summarizing the effects of orchard origin and storage atmosphere on transcriptional responses, metabolic remodeling, and physiological adaptation associated with superficial scald development in ‘Packham’s Triumph’ pear. The model highlights coordinated regulation of stress-responsive genes together with changes in terpenoid, phenylpropanoid, glycerolipid, phytosterol, and sphingolipid metabolism under regular atmosphere (RA), stepwise cooling (SWC), and controlled atmosphere (CA), resulting in differential susceptibility to superficial scald. Genes shown include Chlorophyll A/B binding protein (CAB), Senescence-inducible chloroplast stay-green protein (SGR), Early light-induced protein (ELIP), 9-cis-epoxycarotenoid dioxygenase (NCED), Squalene epoxidase/Squalene monooxygenase (SQLE), Lipase (LPS), Glutathione peroxidase (GPx), Lipoxygenase (LOX), and Glutathione S-transferase (GST).

Superficial scald development in ‘Packham’s Triumph’ pears was strongly influenced by the interaction between orchard origin and storage atmosphere, demonstrating that orchard-specific fruit physiology plays a critical role in the success of postharvest scald control strategies. Controlled atmosphere (CA) consistently provided the greatest suppression of superficial scald, whereas the effectiveness of stepwise cooling (SWC) depended on orchard-specific fruit physiology. Reduced superficial scald incidence was associated with coordinated regulation of stress-responsive genes and remodeling of terpenoid, phenylpropanoid, glycerolipid, phytosterol, and sphingolipid metabolism. The proposed integrated network model (**Figure 6**) illustrates the coordinated interactions among orchard origin, storage atmosphere, transcriptional regulation, and metabolic remodeling underlying superficial scald development and suppression. These findings advance our understanding of the molecular mechanisms underlying superficial scald in pear and identify candidate gene and metabolite biomarkers that may facilitate orchard-specific scald risk assessment and the development of postharvest management strategies.

## Data Availability

The original contributions presented in the study are publicly available. The RNA-seq data have been deposited in the NCBI Sequence Read Archive (SRA) under BioProject accession number PRJNA1513110 and can be accessed at https://www.ncbi.nlm.nih.gov/sra/PRJNA1513110.

## Supplementary material

Supplementary Figure 1 Workflow for candidate gene selection from RNA-seq-derived orthogroups to qPCR-validated genes. Starting from published maturity-related transcriptomic datasets, genes were grouped into orthogroups using PlantTribes2. After filtering based on orthogroup size, gene model quality, phylogenetic validation, and functional relevance, 10 orthogroups were selected. An initial qPCR screen reduced the candidate set to one representative gene per orthogroup, resulting 10 final genes for expression analysis.

Supplementary Figure 2 Preliminary gene expression patterns of stress- and ripening-related genes in ‘Packham’s Triumph’ pears from three orchards at preharvest (-14 d) and harvest (0 d).

Supplementary Figure 3 Differential expression patterns of stress- and ripening-related genes in ‘Packham’s Triumph’ pears from three orchards in response to storage atmosphere (RA, CA, and SWC) and storage durations (-14 d, 0 d, 30 d, and 60 d).

Supplementary File A Ortholog of the maturity related candidate genes were determined based on the gene family phylogeny.

Supplementary File B Primer pairs selected for the qPCR test were underlined. Sequences in < > were excluded from primer design.

Supplementary File C Assembly statistics before post-processing and post processing.
