# Supplementary material for: Orchard and storage-dependent metabolic and transcriptional regulation of superficial scald in ‘Packham’s Triumph’ pears
Source: Front Plant Sci. 2026 Aug 27;17:1885958. doi: 10.3389/fpls.2026.1885958 (PMC13558034; doi:10.3389/fpls.2026.1885958)
Supplement: Supplementary file 1 [file DataSheet1.pdf]

OG170

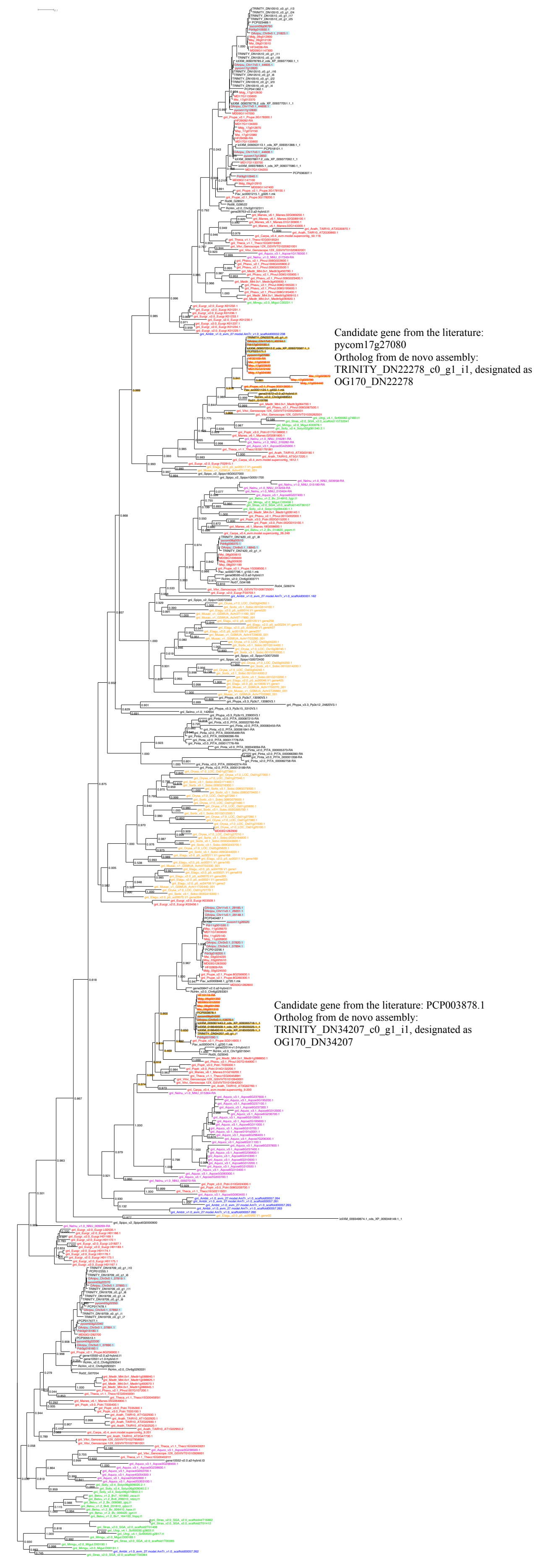
Candidate gene from the literature:
pycom17g27080
Ortholog from de novo assembly:
TRINITY_DN22278_c0_g1_i1, designated as
OG170_DN22278
Candidate gene from the literature: PCP003878.1
Ortholog from de novo assembly:
TRINITY_DN34207_c0_g1_i1, designated as
OG170_DN34207

OG332

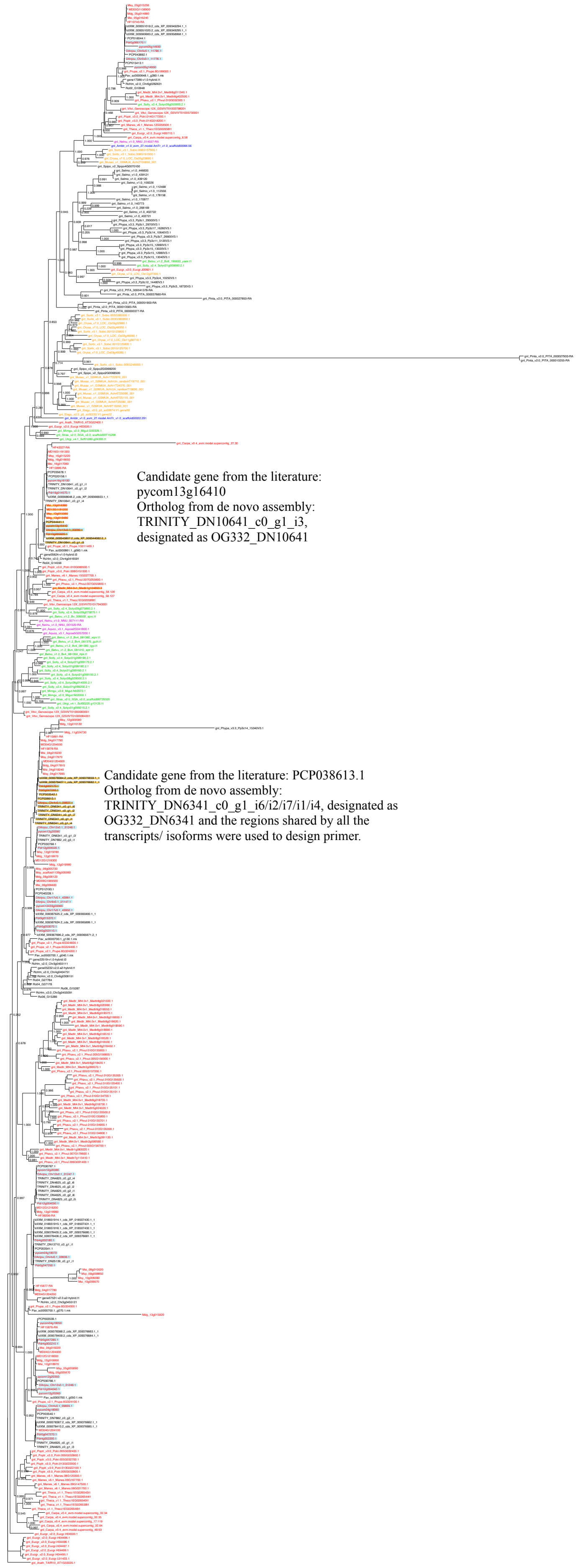

Candidate gene from the literature:
pycom13g16410
Ortholog from de novo assembly:
TRINITY_DN10641_c0_g1_i3,
designated as OG332_DN10641
Candidate gene from the literature: PCP038613.1
Ortholog from de novo assembly:
TRINITY_DN6341_c0_g1_i6/i2/i7/i1/i4, designated as
OG332_DN6341 and the regions shared by all the
transcripts/ isoforms were used to design primer.

OG604

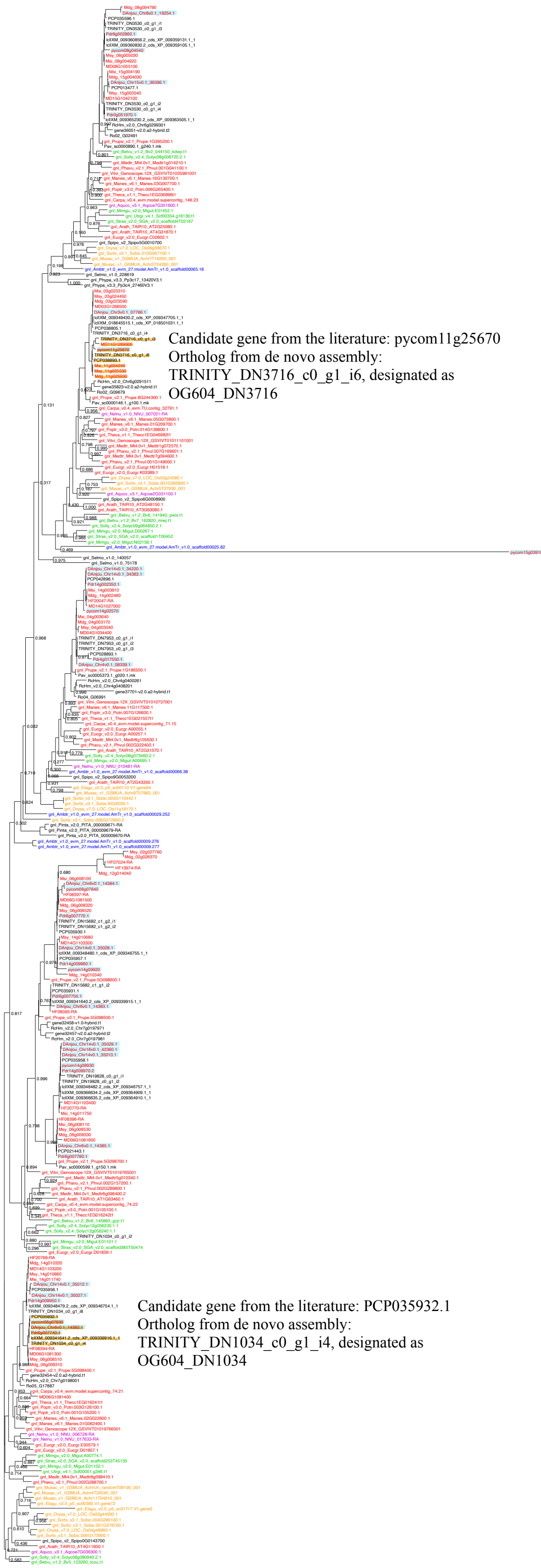
Candidate gene from the literature: pycom11g25670
Ortholog from de novo assembly:
TRINITY_DN3716_c0_g1_i6, designated as
OG604_DN3716
Candidate gene from the literature: PCP035932.1
Ortholog from de novo assembly:
TRINITY_DN1034_c0_g1_i4, designated as
OG604_DN1034

OG611

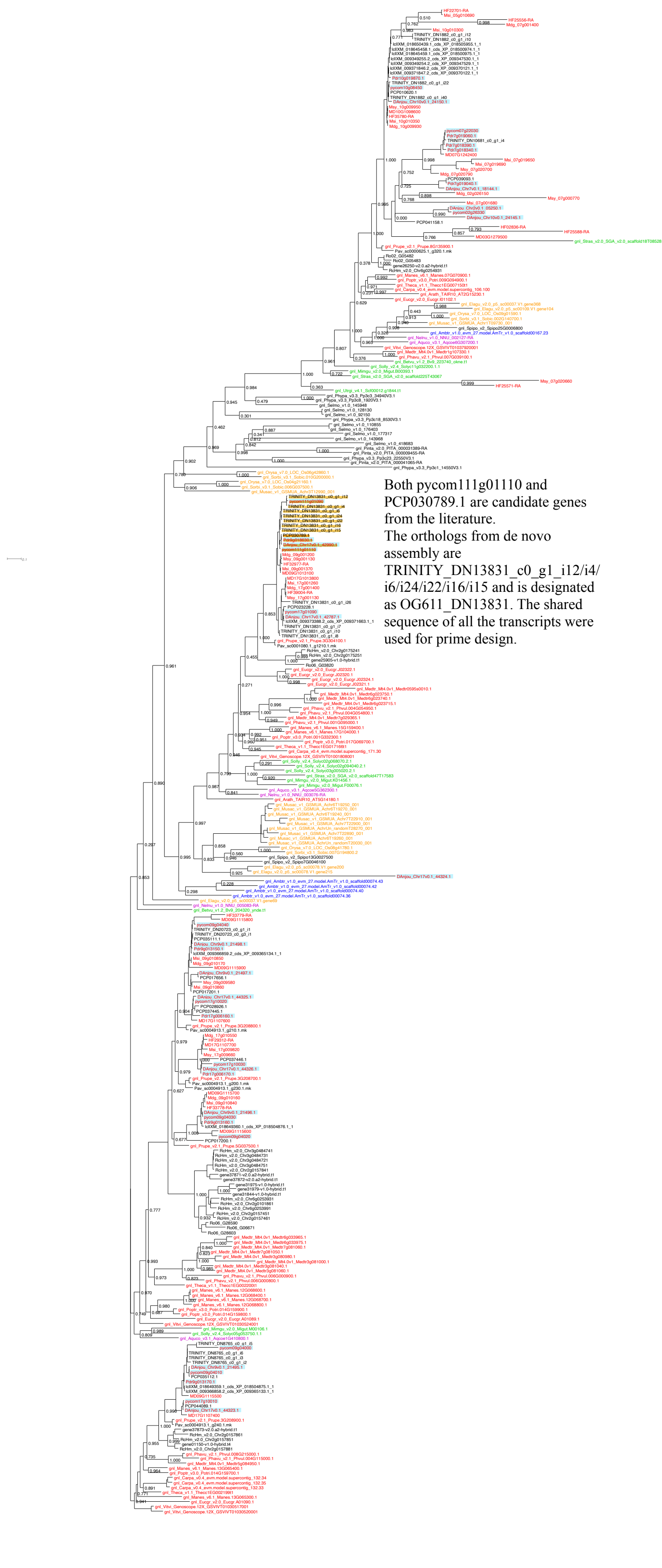

Both pycom111g01110 and PCP030789.1 are candidate genes from the literature. The orthologs from de novo assembly are TRINITY_DN13831_c0_g1_i12/i4/i6/i24/i22/i16/i15 and is designated as OG611_DN13831. The shared sequence of all the transcripts were used for prime design.

OG1014

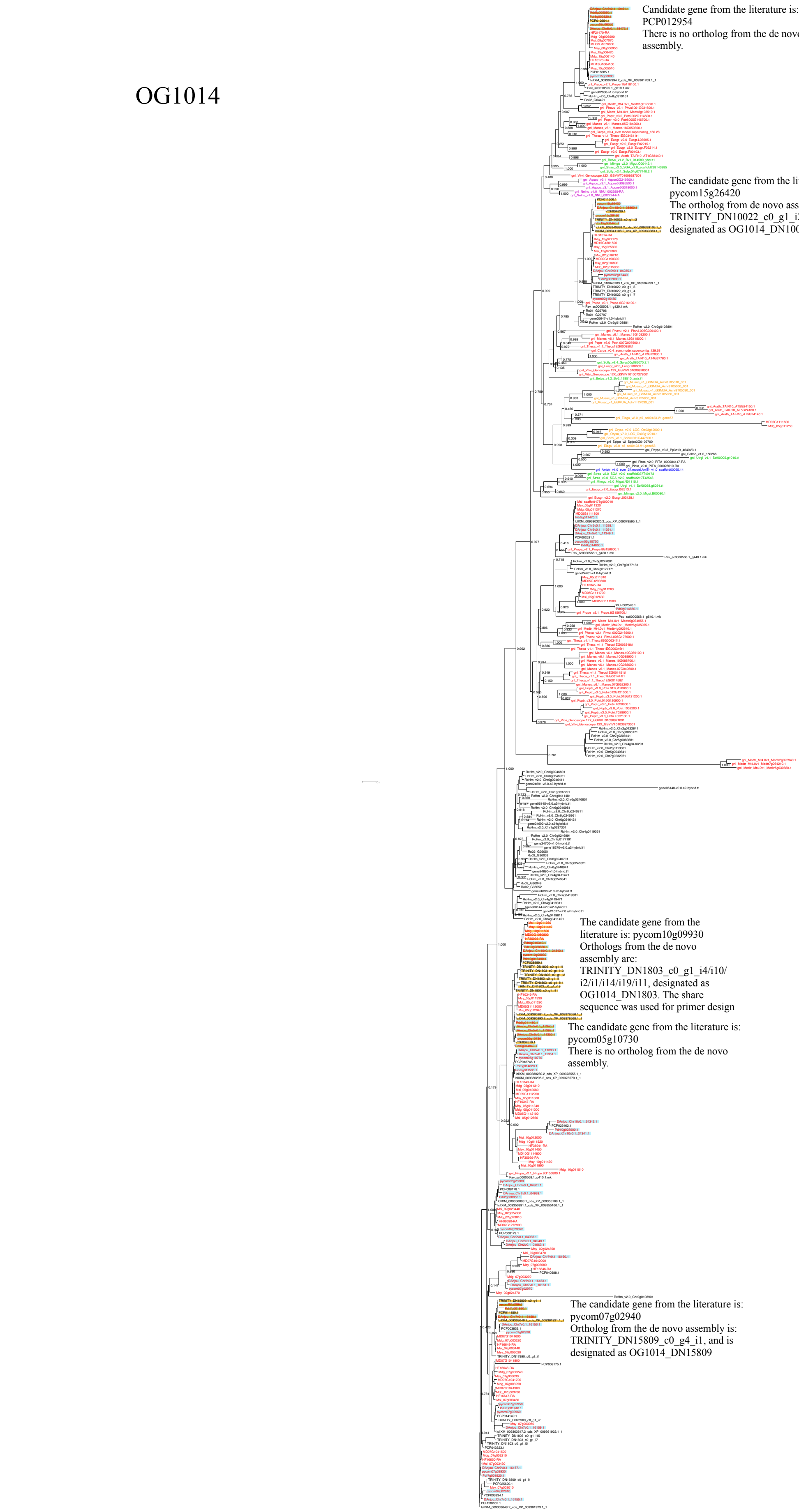

Candidate gene from the literature is: PCP012954
There is no ortholog from the de novo assembly.
The candidate gene from the literature is: pycom15g26420
The ortholog from de novo assembly is TRINITY_DN10022_c0_g1_i2, and is designated as OG1014_DN10022
The candidate gene from the literature is: pycom10g09930
Orthologs from the de novo assembly are: TRINITY_DN1803_c0_g1_i4/i10/i2/i1/i14/i19/i11, designated as OG1014_DN1803. The share sequence was used for primer design
The candidate gene from the literature is: pycom05g10730
There is no ortholog from the de novo assembly.
The candidate gene from the literature is: pycom07g02940
Ortholog from the de novo assembly is: TRINITY_DN15809_c0_g4_i1, and is designated as OG1014_DN15809

OG1184

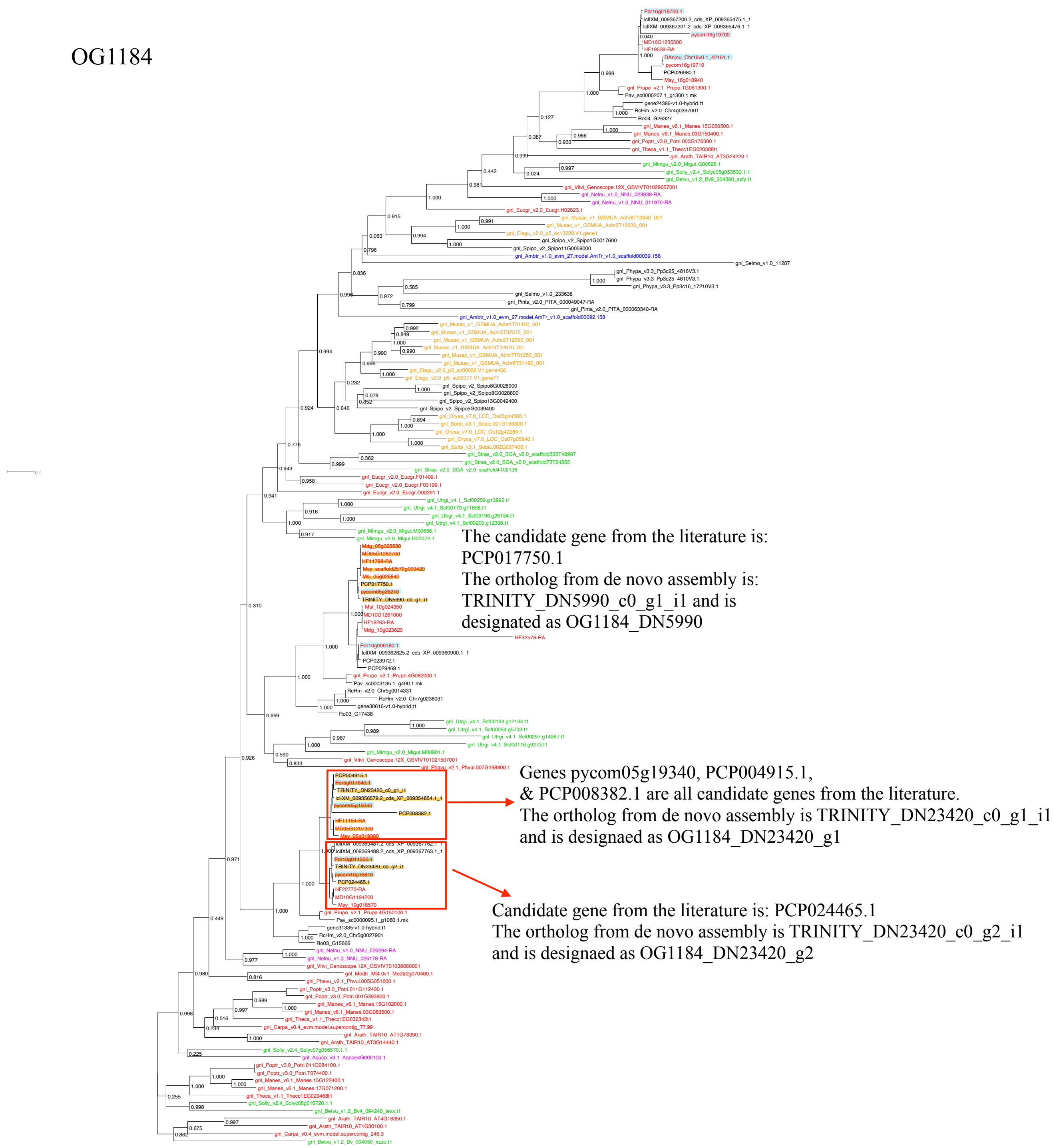
The candidate gene from the literature is:
PCP017750.1
The ortholog from de novo assembly is:
TRINITY_DN5990_c0_g1_i1 and is
designated as OG1184_DN5990
Genes pycom05g19340, PCP004915.1,
& PCP008382.1 are all candidate genes from the literature.
The ortholog from de novo assembly is TRINITY_DN23420_c0_g1_i1
and is designaed as OG1184_DN23420_g1
Candidate gene from the literature is: PCP024465.1
The ortholog from de novo assembly is TRINITY_DN23420_c0_g2_i1
and is designaed as OG1184_DN23420_g2

OG1592

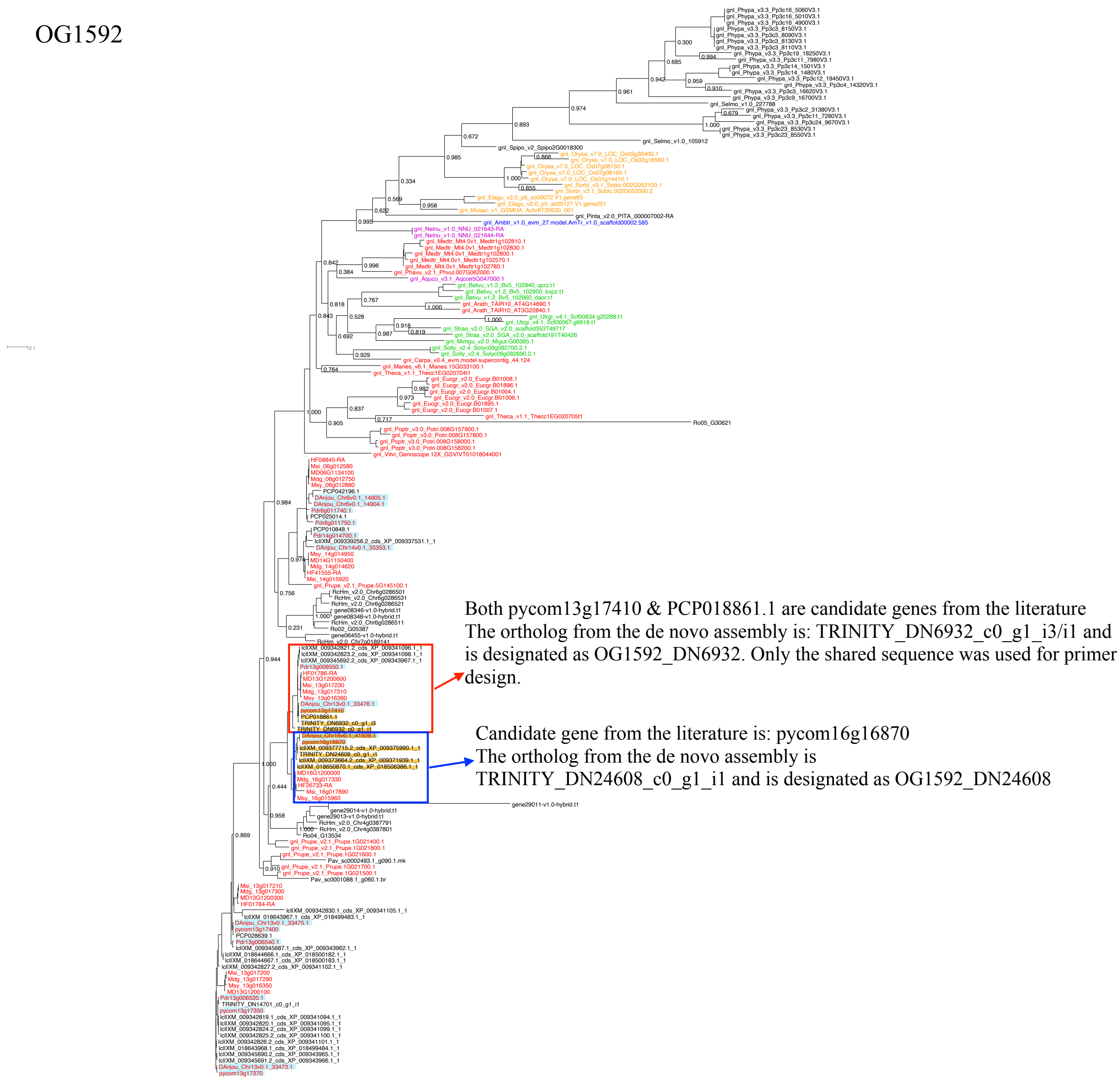

Both pycom13g17410 & PCP018861.1 are candidate genes from the literature
The ortholog from the de novo assembly is: TRINITY_DN6932_c0_g1_i3/i1 and is designated as OG1592_DN6932. Only the shared sequence was used for primer design.
Candidate gene from the literature is: pycom16g16870
The ortholog from the de novo assembly is TRINITY_DN24608_c0_g1_i1 and is designated as OG1592_DN24608

OG2171

Candidate gene from the literature: PCP002056.1
Orthologs from the de novo assembly:
TRINITY_DN3837_c0_g1_i7/i1/i3 and is designated as OG2171_DN3837_i1. Only the shared sequence was used for primer design.

Both pycom15g03390 & PCP015040.1 are candidate genes from the literature.
Orthologs from the de novo assembly:
TRINITY_DN3837_c0_g1_i6/i8/i2 and is designated as OG2171_DN3837_i6. Only the shared sequence was used for primer design.

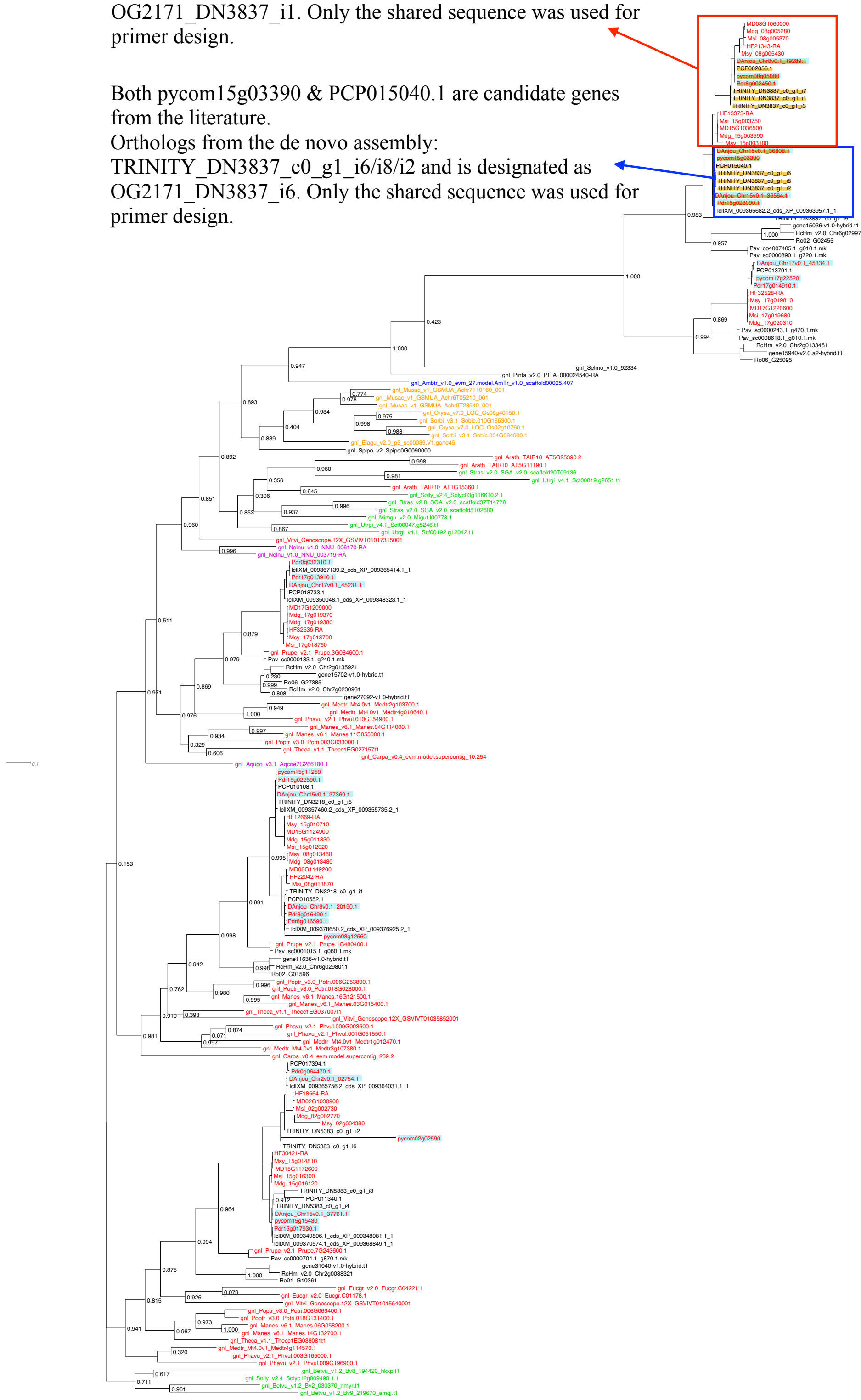

OG2383

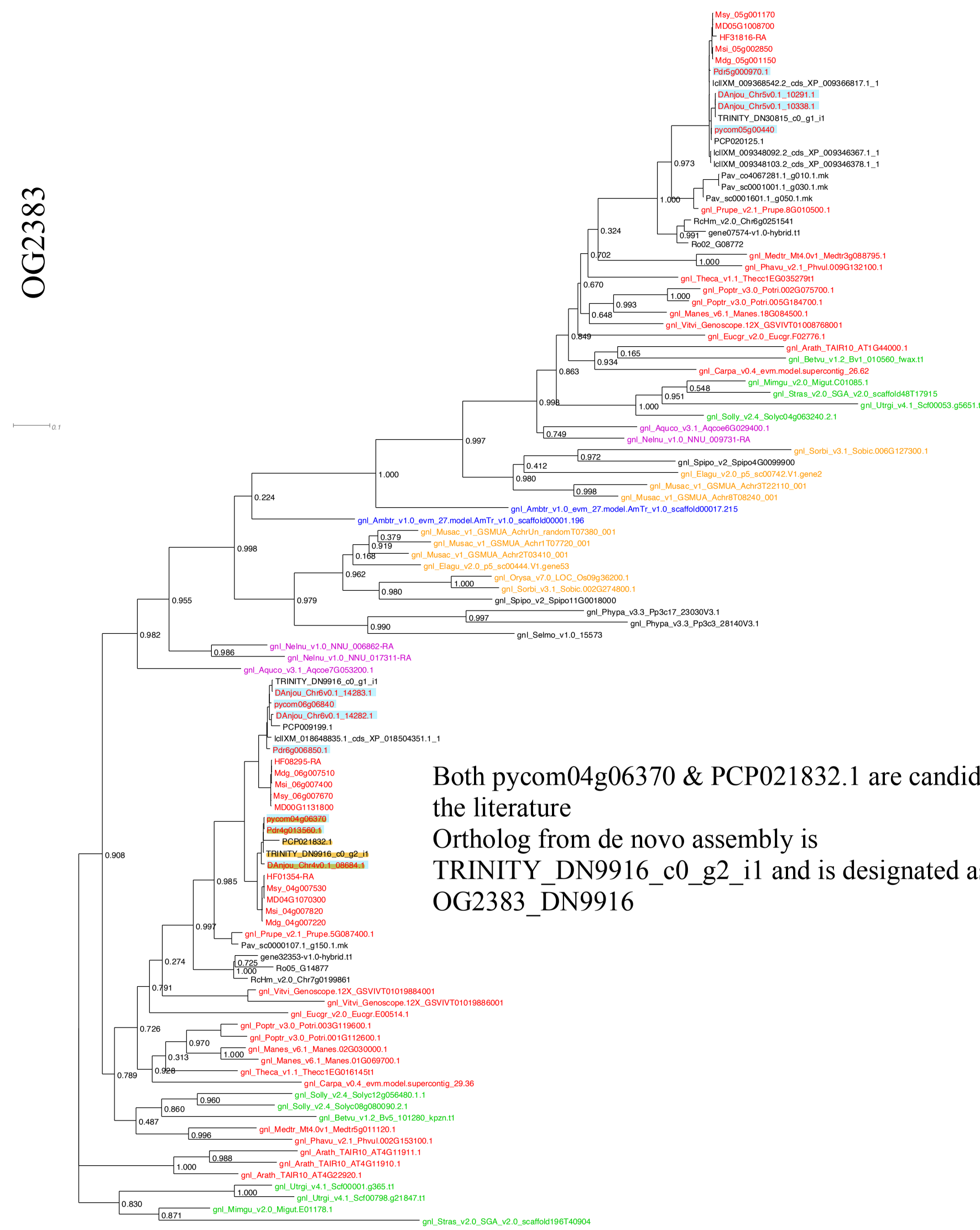

Both pycom04g06370 & PCP021832.1 are candidate genes from the literature
Ortholog from de novo assembly is TRINITY_DN9916_c0_g2_i1 and is designated as OG2383_DN9916

OG3430

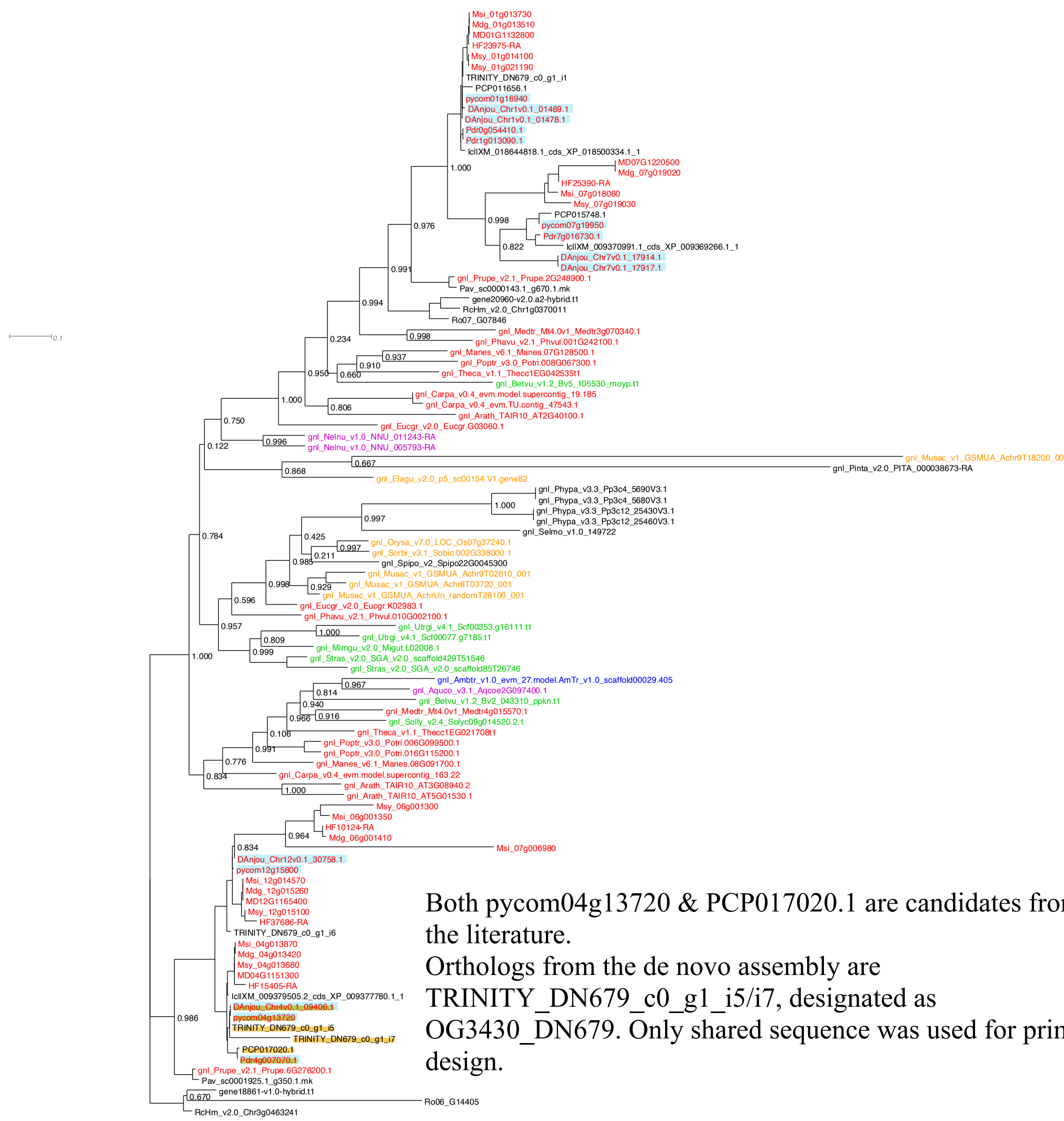

Both pycom04g13720 & PCP017020.1 are candidates from the literature.
Orthologs from the de novo assembly are TRINITY_DN679_c0_g1_i5/i7, designated as OG3430_DN679. Only shared sequence was used for primer design.
