# Supplementary material for: Orchard and storage-dependent metabolic and transcriptional regulation of superficial scald in ‘Packham’s Triumph’ pears
Source: Front Plant Sci. 2026 Aug 27;17:1885958. doi: 10.3389/fpls.2026.1885958 (PMC13558034; doi:10.3389/fpls.2026.1885958)
Supplement: Supplementary file 2 [file DataSheet2.pdf]

Primer pairs selected for the qPCR test were underlined. Sequences in <> were excluded from primer design.

>OG3430_DN679
ATGGCAACAACCACAGCTGCTGCCGCCGCTGCATCCTCATTCATCGGGACACGCCTTCCGACCGACGCGT
ACTCGAACTCGGGACGGGTCCAGGCCCGTTTCGGATTCGGCGCCAAGAAGCCCAAGAAAACAAAACCC
ACTTTCTCAACAGACCGCCCGCTCTGGTATCCGGGCGCCAAGGCTCCTGAATGGCTGGACGGGAGCTTG
GTGGGCGACTA<CGGGTTCGACCCGTTCGGTTTGGGCAAACCCGCGGAGTATTTACAGTACGACTACGA
CGGGTTGGACCAGAATTTGGCCAAGAACTTGGCGGGTGACGTTATCGGGACCCGAACCGAAACCGCGG
ACGTCCAGTCGACGCCGTTTCAGCCTTACACGGAGGTGTTTGGGCTGCAGAGGTTTAGGGAGTGCGAG
CTGATTCATGGAAGGTGGGCCATGCTCGCCACGCTCGGTGCTCTCACTGTTGAGTCCGTCACCGGTATCA
CATGGCAAGACGCCGGAAAGGTGGAGCTCATCGAAGGATCATCCTACTTGGGTCAGCCACTCCCTTTCT
CGCTAACCACGTTGATCTGGATCGAAGTTCTAGTGATTGGCTACATCGAGTTCCAGAGGAACGCCGAGC
TCGACCCGGAAAAGAGGTTGTACCCGGGTGGCAAGTTCTTCGACC>CGTTGGGTCTTGCTGCTGACCCG
GAAAAGAAGGCCACTCTTCAGCTGGCCGAAATCAAGCACGCCCGCCTCGCCATGGTTGCGTTCCTGGGG
TTTGCCGTGCAAGCCGCCGTCACCGGGAAGGGACCACTTAATAACTGGGCTACCCATTTGAGTGACCCG
CTCCACACCACCATTATTGACGCCTTCTCTTCT

> OG2383_DN9916
ATGGGAGCTTTTACTGCTGCTTCTGTGCTTCCTTGGGATTTCAAGCCTTCACTCCCTCTCTCTCATCATAGA
AGCTCTTTGTTTCACTATACAAGAACTCCCAAGAAAAGAAACAGGGCCATAGTTCCTGTTGCGAGGCTAT
TTGGGCCAGCAATATTTGAAGCATCGAAGCTGAAGGTTCTGTTTCTAGGAGTGGATGAAAAGAAGCACC
CAGGGAAGCTTCCAAGAACTTACACACTCACTCATAGTGACATCACCTCAAAGCTCACTCTGGCAATTTC
ACAAACAATCGACAACTCTCAGTTGCAAGGGTGGTACAGCAAGTTGCAAAGAGATGAAGTTGTTGCTCA
GTGGAAGAAAGTTAAGAACAAGATGTCTCTCCATGTTCACTGTCACATAAGTGGAGGTCATTTCCTCTTA
GATTTGTTTGCAAGGCTCAGATACTTCATCTTCTGCAAAGAATTACCTGTGGTTTTGAAGGCCTTCGTCCA
TGGGGACGTCAACTTGTTCAACAGCTACCCAGAACTGCAGGACGCTTCGGTATGGATCTACTTCCACTCG
AGCATTCCCGAATTCAACAAGGTGGAGTGCTGGGGCCCACTCGTCGACGCAGCAGCACCTTCTAGTGGG
TCATCAGGCGGTGCCCACCACCAGGAAAACAACGGGGAGGGGGAGGAGGCAACTTCTCCAAGCAACTG
GGACTTGCCAGAGACGTGTCAAGAGGAGTGCGACTGTTGCTTCACACCACTGACTTCGATTGCATGGTC
CCAAGAGCTTTCCCATGCAAATCAAACTATGGTTGGGACCCACCAGAGCTTTCAGGGGCAAACCCAGGA
AACAAAC

OG2171
>OG2171_DN3837_i1
ATGGCGAGACCGCAACAGCGATATCGAGGCGTCCGACAAAGGCACTGGGGCTCTTGGGTTTCCGAAAT
CCGCCACCCTTTATTGAAGACTAGAATTTGGCTGGGAACATTCGAAACGGCGGAAGACGCGGCGAGAG
CTTACGACGAAGCGGCGAGGCTAATGTGCGGGCCTAGAGCAAGAACAAACTTCCCTTATAACCCGAATG
CGTCTCAGTCCTCGGCCTCGAAACTTCTCTCGGCAACTTTGACAGCAAAACTGCACAGATGCTACATGGC
TTCACTCCAGATGACCAAAACTTCACTGCAAGAGCCAAAAAAGCCGCAAACTTTAGCGTCATCGGGTGC
GAAAATGCCGGATATCAAACCGGAAATTCATCAACCAGTAGCAGAGGAAAATTGGGTTGTGAGGGAAG
TAAAAGTAGAGAGCAGCAGCCAGCAGTTCAAGCCACTTGAAGATGATCACATTGAGCAGATGATTGAG
GAGTTACTGCATTACGGGTCCATTGAGTTATGCTCTGTTGTGCCACCACAAGCTACC

> OG2171_DN3837_i6
ATGGCGAGACCGCAACAGCGATATCGAGGCGTCCGACAAAGGCACTGGGGCTCTTGGGTCTCCGAGAT
CCGCCACCCTTTATTGAAGACTAGAATTTGGCTGGGAACATTCGAAACGGCGGAAGACGCGGCGAGAG
CTTACGACGAAGCGGCGAGGCTAATGTGCGGGCCTAGAGCAAGAACAAACTTCCCTTATAACCCGAATG
CGTCTCAGTCCTCGGCCTCGAAACTTCTCTCGGCAACTTTGACAGCAAAACTGCACAGATGCTACATGGC
TTCACTCCAAATGACCAAAACTTCACTACAAGAGCCAAAAAAACCGAAAACTTTAGTGTCATCGGGCGC
GAAAATGCCAGAGATGATCAGACCGGAAATTCAGCAAGGAGGAGAGGAGAATTGGGTTGTGAGGGAA
GTTAAAGTAGAGAGCAGCAGCCAGCAGTTCAAGCCACTTGAAGATGATCACATTGAGCAGATGATTGA
GGAGTTACTGCATTACGGGTCCATTGAGTTATGCTCTGTTGTGCCACCACAACCTACC

OG1592
>OG1592_DN6932
<ATGGCTGCATCAGCTACCATGCAATCACTTCTAGCTAACTCAGTGGTTTATGGAGCTGGAAGGAGCAG
ATCAGCGAGAGTCATCCACCTTGTTCCTGCTAATAGGTACCGCCACATGGTCGTACGCTCCATGGCCGAG
AATGGTCAGGAGGAGCAACCG>TCTTCTACAATACCAGAGGCATCCAAAACCCCACCACCACCTCCTACA
CCTACTCCTTCTCCTCGTCCTCCCAAGAAGGTTAGCACTAAGTTTTCAGATGTGTTTGCATTCAGTGGGCC
AGCTCCGGAGAGAATCAACGGCAGACTTGCAATGGTAGGGTTTGTTTCAGCTCTGGCAGTGGAACTGTC
CAAGGGCCAGGATGTGTTTACTCAGATCTCCGATGGTGGAGTTTCGTTGTTCCTTGCCACTAGTATCTTG
CTCTCAGTCGCATCCGTGATTCCTCTTTTCAAAGGAGTCAGCGTGGAGTCGAAATCAAACGGGATCATGA
CGTCTGATGCTGAGCTCTGGAATGGAAGGTTGGCTATGTTAGGTTTGGTTGCTTTGGCCTTCACCGAGTA
TGTGAAGGGAGGGACTCTTGTT

> OG1592_DN24608
TCAGATGTGTTTGCATTCAGTGGGCCAGCCCCGGAGAGAATCAACGGCAGACTTGCAATGGTAGGGTTT
GTTTCAGCTTTGGCAGTGGAGCTATCCAAGGGCCAGGATGTGTTTTCTCAGATCTCCGACGGTGGAGTT
TCGTTATTCCTTGGAACTAGTATTTTGCTCTCAGTGGCATCTGCGATTCCTCTATTCAAAGGAGTCAGCGT
GGAGTCCAAATCAAATGGGTTCATGACGTCTGATGCCGAGCTCTGGAACGGAAGGTTGGCCATGTTAG
GTTTGGTTGCTTTGGCCTTCACTGAGTATGTGAAGGGAGGGACTCTTGTT

OG1184
>OG1184_DN5990
ATGGCAACTCTTCCAAAAGCACCCAACTCTAATAGTTGGGCAAAAATGCATCACTCTTTCTCTTCTTCTTC
TTCCCCATCAGTAGTGGATCCTATGGGCTTCCCAAAAAGGTCCATCTTCTTGATGAGCAGCAAAAACCGC
AGAAGTTCTAGTAGCATTCACTGTGCTTTGCAATCTCCCTCGGTTCTTCACTTCCCAAAACAACCCTACAA
CACACCAGTAGTTACCAAGGAAGCTACTCCTGCAAAACCCGGAACACATCAGCTTCCACAGTGGAACTT
ACTACAGAAAGCAGCTGCCATGGCGATCGACATGGTCGAAGGAGCGTTAGTCTCACGCGAGCGCCAAA
ACCCACTTCCCAAAACCTCAGACCCACGTGTCCAAATAGCCGGAAACTACGCTCCGGTGCCGGAACAAG
CCGTCAGCCACGGCTTACCAATTACCGGCACCATACCTGACAGCATTAACGGCGTCTACCTCCGCAACGG
CGCCAACCCTTTACACGAGCCCGTGGCCGGACACCACCTTTTCGACGGCGACGGCATGGTTCACGCCGT
CAGCATCGATTCCGGATCGGCCAGCTACGCCTGCCGGTTCACGGAGACCCAGAGGCTCGTCCAAGAAA
GGGAAATGGGGCGACCCGTTTTCCCCAAAGCCATCGGCGAGCTCCACGGCCACTCCGGCATCGCTCGCC
TCCTCTTATTTTTCGCACGTGGGGCCTTAGGCCTCCTCGACAAAAACCACGGCACTGGCGTCGCAAACGC
CGGCCTCGTCTACCACAACGGCCGCCTCTTGGCCATGTCGGAAGACGACTTGCCGTACCATGTCCGGGT
CACTAAATCCGGGGACCTCGAAACGGTGGGTCGTTACGATTTCGACAGCCAGCTGGGGTCCACAATGAT

CGCACACCCGAAGGTCGACCCGGAATCCGGCTCCCTCCACGCGCTCAGCTACGATGTTGTTCAGAAGCC
GTATTTAAAATACTTCCACTTCAGCCCCAACGGCGCGAAATCCCCGGACGTGGAGATCCCATTGGCAGG
GCCCACCATGATGCATGACTTTGCGATCACCGAGAACTACGTGGTGATCCCGGACCAGCAGGTCGTGTT
CAAGCTCGGAGACATGATCACGGGTGGGTCCCCCGTGATCTACGACAAGAGCAAGATGTCGCGGTTCG
GAATTCTTAAGAAGAATGCCGACAACGCCGACGACATCATCTGGGTCGACTCTCCCGACACGTTCTGCTT
CCACCTCTGGAACGCGTGGGAGGAGCCCGAGTCCGATGAGGTCGTCGTGATCGGCTCCTGCATGACGC
CGCCCGACTCGATCTTCAACGAGTGCGACGAGAACTTAAAGAGCGTGCTGTCCGAAATCCGGCTCAATT
TGAAAACCGGCGAGTCGAATCGGCGGGCTATTTTGTCTGAATCGGATGATGTGAATTTGGAGGCGGGA
ATGGTAAATAGGAACTTACTCGGGAGGAAAACGCGGTTCGCTTACCTCGCAATTGCCGAACCCTGGCCT
AAAGTTTCGGGTTTTGCTAAAGTTGATCTTTCTACAGGGGAGGTGAAAAAGTTTTTTACGGCGAGAAA
ATGTACGGCGGTGAGCCCTTTTTCGTGCCGAGCACGGAAGAGGGCGCGTCGGAGGACGACGGTTACAT
TCTGACGTTCGTTCACGACGAGAAGAACTGGAAGTCGGAGCTTCAGATTGTTAACGCGGTCAATATGAA
GTTGGAGGCTACGGTAAAATTACCTTCAAGGGTGCCCTACGGGTTTCATGGGACGTTTATAGAATGGAA
GGACTTGGTAAATCAAGCA

>OG1184_DN23420_ g1
CACGTCCAAATCACCGAAACAGGGGACCTCAAAACGGTCGGCCGCTACGACTTCGAAGGGCAGCTGAA
GTCCACAATGATCGCCCACCCGAAGGTGGACCCCGCCACGGGCGAACTCTTCGCGCTTAGCTACGACGT
CGTTCAGAAGCCATACCTCAAATACTTCAAATTCTCCCCAAGCGGCGACAAATCCCCGGACGTCGAAATC
AACCTCGAGCAACCCACCATGATGCACGACTTCGCCATTACCGAACGACACGTCGTAATCCCAGACCAGC
AGGTGGTGTTCAAGCTGCAGGAGATGATCGGCGGTGGGTCCCCCGTAATTTACGACAAGAACAAGATG
TCGCGTTTCGGGATCCTCGACAAGAATGCCAAGGACGCGTCGGGAATCCGCTGGGTAGACTGC

>OG1184_DN23420_g2
CAAATCGCCGGAAACTTTGCTCCGGTGCCGGAGCACCCCGTCGAACACTATCTACCGGTGACCGGAAAA
ATCCCCGACTCAATCCAAGGCCTCTACGTCCGAAACGGCGCCAACCCACTTCACGAGCCGGTCGCCGGT
CACCACCTCTTCGACGGCGACGGCATGGTCCACGCCCTCCAGTTCAAAGACGGAACCGCCAGCTACGCT
TGCAGGTTCACCGAAACCAGCCGTATCAGCCAAGAACGTGACCTCGGCCGTCCCCTGTTTCCCAAAGCC
ATTGGCGAGCTCCACGGCCACTCCGGCATCGCCCGCCTCCTCCTTTTCTACGCCAGAGGGATGTTCGGAC
TCGTCGACCCCACCCACGGCGTCGGCGTCGCTAACGCCGGCCTCGTC

OG1014
>OG1014_DN1803
ATGGATTACCAGTATGTTCTTGGTGGGGTGTTGGTTTCTCTGCTGGGCTCTGTTTTCTTCATGATCATCAA
CACCACTCTCAACGGCGAGAAGAAGAAGGTGGTCGAGAAAGTTTCCAACGGCGTTAACGGAAATGGTT
ACGTGAGGATGCCGTCCCAGAACGGTAATTTTCAACCGGAGAGTGCCAGTGGCACGGACGTTGTCATTG
TCGGTGCCGGAGTTGCCGGTGCTGCCCTTGCTTACACTCTTGCGAAGGACGGACGCCGGGTACATTTGA
TCGAAAGAGACTTGAGTGAGCCAGACAGAATTGTTGGTGAACTATTGCAGCCTGGAGGCTATCTCAAAT
TGATTGAGTTGGGTCTTGAAGACTGTGCAAATGAGTCCATTGACGCTCAAAAAGTATTTGGTTATGCTCT
CTACAAAGATGGGAACGACACAAAACTGTCTTATCCCTTGGAAAACTATCCTTCAGATATCGCCGGAAG
AAGTTTCCACAACGGACGTTTCATTCAAAAAATGCGTGAAAGGGCTACAGCTCTTAAAAACGTGACACT
AGAACAAGGAACTGTGACAACCCTAATTGAAGAAAAGGGCACCGTCAAGGGTGTGATGTACAAGAACA
AGGCTGGAGAGGAGATGAGATCAAATGCTCCATTAACAATAGTATGCGATGGGTGCTTTTCAAATCTCC
GCCGCAATCTCTGTACTTCCAAGGTTGACAATCCTTCTTGCTTCGTCGGCTTGATCTTGGAGAACTGTGAC

CTTCCTCATGCAAACCACGGACACGTGATTTTGGGAGACCCATCACCTATCTTATTTACCCTATTAGTGG
TACCGAAGTTCGTTGTTTGGTAGATGTTCCCGGCACAAAAGTACCTTCAGTAGCTAATGGTGAAATGGCC
AAGTACTTGACAAATGTGGTTGCTCCTCAGGTTCCCCCTCAGCTCTCGAAGGCTTTTCTAGCATCAGTTGA
GAAAGGAAACATCAGAACAATGCAAAACAAAAGCATGCCTGCTCATCCTCAACCCACTCCCGGTGCAAT
TTTGTTAGGGGATGCATTCAACATGAGACATCCTTTGACCGGAGGAGGAATGACCGTGGCTCTTTCCGA
CATTGTTCTTCTAAGGGATCTTCTTAGACCCCTAAATGATCTCAACGACGCACCAGCTTTGTGCGAATATC
TAGAATCATTCTACACACTTCGCAAGCCTGTGTCATCTACCATAAACACATTGGCCGGTGCCTTGTACAA
GGTGTTTTGTGCATCACCTGATCCGGCAAGACAGGAAATGAGGGAAGCTTGTTTTGATTATTTGAGCCTC
GGAGGCATATGTGCAAGGGGACCAGTATCTCTACTCTCTGGCCTTAATCCTCGTCCGATCCACCTTTTTCT
CCATTTCTTTGCCGTGGCTGTCTATGGAGTAGGTCGCTTAATGATTCCATTCCCTACGCCTAAACGTGTAT
GGCTCGGGACCAGATTGCTCTTGGGTGCATCAGGAATTATATTCCCCATTATTAAAGGTGAAGGAGTTA
GACAAATGTTCTTTCCTGCAACAATCCCAGCATATTACAGAGCTCCTCCTCTTAAA

There are 7 transcripts share high similarity, design primer at the shared region.

>OG1014_DN1803_shared
ATGGATTACCAGTATGTTCTTGGTGGGGTGTTGGTTTCTCTGCTGGGCTCTGTTTTCTTCATGATCATCAA
CACCACTCTCAACGGCGAGAAGAAGAAGGTGGTCGAGAAAGTTTCCAACGGCGTTAACGGAAATGGTT
ACGTGAGGATGCCGTCCCAGAACGGTAATTTTCAACCGGAGAGTGCCAGTGGCACGGACGTTGTCATTG
TCGGTGCCGGAGTTGCCGGTGCTGCCCTTGCTTACACTCTTGCGAAGGACGGACGCCGGGTACATTTGA
TCGAAAGAGACTTGAGTGAGCCAGACAGAATTGTTGGTGAACTATTGCAGCCTGGAGGCTATCTCAAAT
TGATTGAGTTGGGTCTTG

>OG1014_DN10022
AATGCACCCATGGTGGCTGTTGATCCGTACGTTCTCTTGACCTTCTTCGCCTCCCTCTTAGGTTTCCTCGTT
CTGTCTGTTCTCCGACGCCGTAATTACAGCAAAGCCAAGAGCAAGGCTCAAAACAACAATACGAACGTT
CCAGAAAAAAGTATCAACTGCTTCGATGACGGAGAAGTCTCGCCAGTTCACGGCTCTGGCACCGACGTC
ATCGTTGTCGGTGCGGGCGTTGCTGGCGGTGCTCTCGCCCTTACCCTCGCTAAGGAGGGAAGACGTGTT
CATGTGATTGAAAGAGACCTAACGGAGCCGGATCGAATTGTCGGTGAGCTTCTCCAGCCGGGAGGGTA
CCTGAAATTGATTGAGTTGGGACTTGAAGATTGTGTGGAGGAAATTGATGCTCAGCGAGTGGTTGGATA
CGCTCTTTTTAAGGATGGGAAGAACACTCGGCTGACCTATCCTCTGGAACAGTTCCACTCCGATGTGGCG
GGGAGAAGTTTCCACAATGGCCGATTCATACAGAGGATGAGAGAAAAAGTTGCCAAGCTTCCCAATGTA
CAACTGGAGCAAGGTACTGTCACATCCCTGCTTGAAGAAAATGGAACAATTAAAGGGGTTCAATACAAA
CTTAAGGATGGTCAAGAACTTAAAGTTTACGCTCCTCTTACAATTGTTTGTGATGGTTGCTTCTCAAATCT
GCGCCGCACTCTTTGCAAGCCTCAGGTAGAAGTACCTTCTTGCTTTGTAGGACTAATCTTGCAAAATTGC
AAACTCCCATTTGCAAATCATGGGCATGTTATTCTAGCGGATCCCTCTCCGATCCTGTTTTATCAAATCAG
CAGTACAGAAGTCCGTTGTTTGGTTGATGTACCCGGGCAAAGGGTACCTCCAATTGCCAATGGTGCGTT
GGCCAATTATTTGAAGAATGTGGTGGCTCCACAGGTTCCACCTGAACTTTATGATGCCTTCATTGCTGCA
ATTGATAAAGGAAATATTAGAACAATGCCAAACAGAAGCATGCCTGCCAGTCCACATCCAACTCCAGGA
GCTCTTTTGCTGGGCGATGCTTTCAATATGCGTCATCCTTTAACTGGCGGAGGAATGACTGTGGCACTGT
CAGATATTGTCGTGCTCAGGGATCTTCTTAAGCCATTGCATGACCTACATGATGCAGCTTCATTGTGCAC
ATACCTAGAATCGTTTTATACCTTACGAAAGCCGGTTGCATCTACAATAAATACCTTAGCAGGTGCCCTAT
ACAAGGTGTTCTCTGCCTCCCTTGATGAAGCAAGGATAGAGATGCGCCAAGCATGTTTTGACTATCTCAG
CCTTGGAGGTGTCTGTTCAACGGGACCAGTGGCTTTGCTTTCTGGTCTAAACCCTCGTCCGTTAAGTTTA

GTCCTTCACTTTTTTTCTGTGGCGGTATATGGTGTTGGTCGTTTGCTACTACCATTTCCTTCAGTAAAACGC
ATGTGGCTTGGAGCTAGATTACTTTTGAGTGCATCGGGTATTATATTCCCCATCATCAAGGCGGAAGGA
GTAAGACAAACCTTCTTTCCTGCTACTATTCCAGCTTATTACAGAGCTCCTCCTGCTAAG

>OG1014_DN15809_c0_g4_i1
ATGGTTTACGAGTATGTTCTTGCTGGTGTCTTGGTTTCTCTGTTGGGTTCGGTTTTCTTTGTGATCATAAA
CACTACTTTGAGCGAAAATAAGAGGGACGTTGCAATTGTGTCAGAAAACGGTGTTTGTTTGGCACCGGA
GATTGAGAAAAGTACCGACGTTGTCATTGTTGGTGCCGGAGTTGCTGGTGCTGCTCTTGCTTACACTCTT
GGAAAGGAAGGACGACGTGTACATGTGATTGAAAGAGACTTAAGTGAGCCAGACAGAATTGTGGGTG
AACTATTGCAGCCTGGAGGCTATCTCAAATTGATTGAATTAGGTCTTGAAGATTGTGCAAATGAG

OG611
>OG611_DN13831
ACTCAGTTTGTTATGGCAGCATGGGCGAGCTTCTGCTTCCTACTATCGCTTGCAGCACTTTCACCTCATCG
AGCGCACGGCTCCACTTGGGATTCACTATTCGGCCGCAAACACGGTGCTGCGGCCGCCCCGACAGTTGG
TATATGCGCGTCGTCGGTGGTTGTGTACGGCTACCCATGTCAGGAGTTTGACGTGACAACTCAAGATGG
TTATATATTGAGTGTGCAAAGGATTCCGGAAGGTCGCGGGAAAGGCAGTGGTGGCGGCGGGATTAAG
AAGCCACCGGTCTTAATTCAGCATGGTGTCCTTGTGGATGGAGTCACCTGGCTCTTGAATTCTCCGGACA
GAAATCTGCCGTTGATTCTGGCGGACGACGGATTTGATGTGTGGATTGCTAACACCAGAGGGACCAGGT
TCAGCAGGCGACATACTTCCATGGATCCCCG<CGATCCGAAATTTTGGAATTGGTCTTGGGATGAACTG
GCGGCTTTTGATCTACCAGCAGTGTTTGATTTCGTGTATGGAAAGGCAGGACAGAAGATCAATTATGTT
GGCCATTCC>ATGGGAACTTTATTTGTATTGGCATCGTT<ATCAGAAGGGAAACTGGTGGACCAGATGA
AATCAGCAGCCTTGCTAAGCCCCATTGCTTATTTGAGCCACATGAACACTGCACTTGGTGTGGCTGCTGC
CAAAGCCTTTGTTGGTGAGATCACT>ACGCTTTTCAGACTTGCGGAGTTCAACCTGAAAGGGGACCCAG
CGGCTCAATTTCTGAAAGCTCTATGCGCTTATCCATGGGTTTACTGCTATGACTTACTACAAGCACTTGCT
G<GCAAAAACTGCTGTCTCAATTCTTCCACCGTCGATCTCTTCTTGGAGAATGAGCCTCAGTCAACATCCA
CCAAGAACATGGTGCACTTGGCTCA>AACTGTACGAGATGGGAAGTTGGCAAAATATAACTACGGCAG
ACCAGACTTAAACTTGATGCATTATGGCGGATTTAACCCTCCAGTTTACAACCTCTCCAACATTCCCCATG
ACCTTCCTCTGTTCCTCAGCTACGGCGGCCAAGACTCGCTCTCCGATCTTCGTGACGTGGCGCGCTTACT
GGATAGTCTAAAACTTCACGATGTGGGGAAGCTCACTGTTCAGTACATAGAGAATTATGCACATGCAGA
TTTCATCATGGGGTTGAATGCTAAGGACATTGTGTATCGTCAAGTGACTGCATTTTTCAAGCAGCAACAC

>OG604_DN1034
<TGCATCTGCAATTCTACTCGTTTTCTCTTCCGACGAAAACTCACCGTTGCCGCTTCATCTGCGTCTCAGC
GTCTGCAAAAGCACTTCTCCGCCGATTCCTTGCAGACCCTTTTGCCACGGTCTCTGTTTTCGCCGTCTATC
AAGGTCGAGTTTTCAAGCCCGGTTGCATCTACTTTGCTGTTGAATCGTACA>ATGGCTGGCCAGTCCGAG
AGTAAATCAATCCATGACTTCACTGTCAAGGATGCCAAGGGTAATGATGTTGATCTCAGCACCTACAAA
GGGAAGGTCCTTCTGATCGTTAACGTTGCGTCGCAATGTGGCTTGACTAATGCAAACTACACAGAGTTG
GCTCAGTTATATGAGAAATACAAAACTCAAGGTTTGGAAATCTTGGCATTCCCATGCAACCAGTTTGGAG
CTCAGGAGCCAGGGACAAATGACGAGATTGTCGAGTTTGCCTGCACTCGCTTCAAGGCTGAGTATCCCA
TCTTTGACAAGGTGGATGTGAATGGCGACACTGCCGCTCCGATTTACAAATT<CTTGAAGTCGAGCAAG
GGTGGACTCTTTGGCGACAACATCAAGTGGAACTTCTCCAAGTTCCTGGTTGACAAAGAAGGGAAAGTC
GTTGACCGTTATGCCCCTACAACTTCTCCTCTTAGCATTGAGAAGGACGTGAAGAAACTGCTGGGGGTC
GCA>

>OG604_DN3716
ATGGGTGGCTTCCAATCTGTCTCTGAGCAATCCATCCATGAATTCACAGTGAAGGATAGCAGAGGGAAG
GAGGTGGACCTCAGCGTTTACAAGGGGAAGGTCGTCCTCGTCGTCAACGTTGCTTCCAAATGTGGGTTT
ACTGATACGAATTATACCCAGTTGACTGAGATTTACAGCAAATACAAAGAGAAAGGTTTTGAGATCTTG
GCATTTCCGTGCAATCAGTTTCTAAAGCAAGAGCCCGGGACAAGTCAGGATGCTGAACAATTTGCATGT
ACAAGATACAAGGCTGAATATCCAATATTCAAGAAGGTACGTGTCAATGGGCCAGATACCGAACCTGTT
TACAAATTCCTCAAAGCAAGTAAATCTGGATTTCTGGGGAATAGCATAAAGTGGAACTTCACCAAGTTCT
TAGTTGACAAGGATGGACATGTAATTGAACGGTACGCCCCAACCACCTCCCCCTTGAGCATTGAGGCCG
ATATCAAGAAAGCGCTAGGGGAGGCA

>OG332_DN10641
<GTTCAAATATTGCTTCCAGAACTTAAGTCTTTGTGCGATAAAACTATCAACGAGTTTGACACCTTTGAGG
ATGTACTTAACCTCTATGACGGAGGTATCAAGCTACCAAATGGACCTGCTCTCAAGAAAATACGGGAGT
GCATCCCTTGGGAGCTGCTTAAGGAACTGATTCGCTCAGATGGCGAGCGATTTCTCAAGTTTCCAATGCC
TGATGTGATTAAAAGAGATAGGTCTGCTTGGAGGACAGACGAAGAGTTTGCACGAGAAATGCTAGCAG
GGGTGAACCCTGTTAACATCACCCGCCTC>GAAGAGTTTCCCCCAGTCAGTAAGCTAGACCCTGAAGTAT
ATGGGAATCAGAACAGTTCGATACGAGAAGAGCACATAAAAGGCAACATGAATGGCCTCACTGTTGAA
GAGGCAACTGAAAAGAACAGGCTATTCATATTAGATCATCATGATGCATTAATGTTATACCTGAGGCAA
ATAAACTCAATGGACACAAAGACGTATGCCACAAGAACTCTTCTCTTGCTGCAAGAAGACGGGACGTTG
AAGCCTTTGGCAATTGAGCTAAGCTTACCACATCCACAAGGGGACCGTCATGGGGCTGTCAGCAAAGTT
TTCACTCCACCAGGACAAGGCATCGAGGAGTCAGTGTGGCAGCTTGCCAAAGCTTATGCTGCTGTAAAC
GATTCTGGCTATCATCAA<CTTATCAGCCACTGGTTAAACACACATGCTGTGATAGAGCCATTTGTGATT
GCAACACATAGACAGTTGAGTGTGCTTCACCCGATACATAAGCTTTTGCATCCGCACTTCCGGGACACAA
TGAACATAAATGCCCTGGCCAGGCAAATCCTCATCAATGCCGGTGGGGTGCTTGAGAAAACAGTCTTCC
CAGGTCGATTCTCCATGGAAATGTCAGC>TGTTATTTATAAGAGCTGTGTACTTACCGAGCAGGCATTAC
CTGCTGACTTGCTCAAGAGAGGAATGGCAGTTCCAGATACGACTTGTCCTCATGGC>

>OG332_DN6341
TCGATTCACCTCACCACCCACCTTTTTTCACAAAACCTTAATCAGAAGAACAAAATGCTGCAGAATATCGT
TGAGAAGCTCACAGGTCACCAGCAAAATGGTAGTCATGGAAAGATCGATGGGACTGTGGTGCTGATGA
AGAAGAATGTTTTGGACTTCAACGACTTCAACGCTTCTGTTCTCGATCGCGTGCATGAGTTGGTCGGCCA
AAGGGTTTCTCTGCAGCTCATCAGTGCCGTTCGTGCTGATTCCGAAAATGGATTGAAGGGGAAGCTTGG
AAAGCCAGCTTATTTGGAAGACTGGATTACCACAATCACACCTTTAACAGCAGGAGAGTCTGCATTCAA
GGTTACATTTGACTATGAAGAGGAAGTAGGAGTTCCGGGAGCATTCTTAATCAAAAACAACCACCACAG
TGAGTTCTTCCTCAAGACCGTCACACTCGAAAATGTTCCCGGTGAGGGCCGTGTACACTTCGTTTGCAAC
TCTTGGGTCTACCCTACTGAAAAATACACCAAAGACCGTGTTTTCTTCGTCAACAAGACTTATCTTCCAAG
CGAGACACCATTGCCATTGCGAAAATACAGAGAAGAAGAGCTAGTACACTTGAGGGGAGATGGAAAAG
GAGAGCTCCAAGAATGGGAAAGGGTTTATGACTATGCTTACTACAATGATCTGGGGAAACCTGATAAG
GGTGCCAAATACGTCCGTCCTGTTCTTGGAGGGTCTAGTGAGTACCCTTACCCTCGCCGTGGAAGAACT
GGCCGACCACCAACTAAGACAGATCCTAACAGCGAGAGTAGTCTGCCGCTTATTCAGAGCTTAGACATC
TACGTTCCTAGGGATGAAAGATTTGGACACTTGAAGCTGTCAGACTTCCTTATTTATGCCCTGAAATCCA
TAGCTCAGTTCATTAAACCGGAGCTGGAATCCTTGTTTGACCAAACTCCTAGCGAGTTTGACAGCTTCGA
AGATGTGCTTAAGCTCTACAAAGGAGGCATTCCGTTGCCTGAAGGTTTATTGAAGGATATTGGAGACAA

CATCCCTGCGGAGATGCTGAAGGAAATTTTCCGAACTGATGGTGCACAATTGCTCAAATTCCCAACGCCT
CAAGTGATCAAAGAGGATAAATCTGCTTGGAGGACCGATGAAGAATTTGCTAGGGAAATGCTGGCTGG
AGTGAACCCTGTCAACATTGCTCGTCTCCAAGTATTTCCGCCAGCAAGCAAGCTAGATCCAAAAGTTTAC
GGTGATCAAAACAGTACAATAACCGAAGAACATATAAAGAACAACTTGGATGGACTAACAGTGGATGA
GGCACTCAAGAAAAACAAGTTATTCATACTAGACCATCATGATTCACTGATGCCGTACCTGAGGCGGAT
AAACTCAACTTCGAACAAGATCTACGGTAGTAGAACTCTGCTGTTCTTGCAAAATGATGGAACTTTGAAA
CCATTGGTGATCGAGTTGAGCTTGCCTCATCCTGACGGAGATCAATTTGGTTGCATTAGTAATGTCTACA
CACCAGCTGAACAAGGTGTGGAAGGTTCCATATGGCAACTGGCTAAAGCTTATGTGGCTGTCAATGACT
CCGGAGTCCATCAACTCATCAGCCATTGGTTGAACACTCATGCAGTGAGTGAGCCAGTTGTAATTGCCAC
AAACAGGCAACTAAGCGTGGTTCACCCGATTTACAAACTTCTGCATCCTCACTTCCGTGACACGATGAAC
ATCAATGCATTTGCGAGGCAAATCCTCATCAATGCTGGTGGTATTCTTGAGACAACAGTTTTCCCAGCTA
GGTATGCCATGGAGCTGTCATCAGTTGTGTACAAGGATTGGAATTTCACTGAGCAACCCCTCCCTGCAG
ATCTCATCAAAAGAGGAGTGGCAGTTAAGGATAAAAATTCTCCACACGGTCTTCGCCTACTGATCGAGG
ACTACCCTTATGCTGTTGATGGGATTGAAATCTGGTTTGCAATAAGAACATGGGTTGCAGACTACTGCTC
TTTCTACTACAAGACCGACGATATAATCCAGAAAGACGCCGAGCTCCAATCCTGGTGGAAGGAGCTAGT
AGAGGAAGGCCACGGTGACAAAAAAGACGAGCCCTGGTGGCCAAAATTGCAGACTCTCGAAGAGCTA
GTTGAAATCTGCACAACTATCATATGGACTGCTTCTGCCCTCCATGCAGCTGTCAACTTCGGGCAGTACC
CTTACGCCGGATACCTCCCCAACCGCCCTACTATAAGCCGAAAATTCATGCCCGTAAAGGGAACTGCTGA
ATACGAAGAGCTCAAGTCCAACCCCGACAAAGTGTTCTTGAAAACAATCACTGCTCAGCTGCAGACACT
GCTTGGCATTTCCCTCATTGAAATTCTGTCAAGGCATTCTACTGACGAAGTGTACTTGGGGCAGCGAGAC
ACGCCCGAGTGGACAGCAGACGCTGCACCATTGGAAGCATTCGACAAGTTTGGAAAGAAACTGGAGGA
GATTGAGGAGAGGATTACAAGTATGAACAATGACGAGAAGTTGAAAAACCGAGTTGGACCGGTGAAG
GTGCCTTACACTTTGCTGTTTCCTACTAGCGGAGGGGGACTTACTGGCAAGGGAATTCCCAACAGTGTCT
CAATT

>OG170_DN22278
TACAAGAAGATGGTTGTCAAAGTATATGGTCCAGTAATGGCAGCCTGCCCCCAGAGGGTGATGGTTTGC
CTTTTGGAGAAAGGAGTGAACTTTGAAATTGTAGATGTTGATCTTGAGGCAGGAGAGCACAAGCGACC
CGAGTTTCTCACCCGTCAGCCGTTTGGGCAAGTTCCAGTTGTAGAAGATGGTGACTTAAGGCTCTTTGAA
TCTAGGGCCATCATAAGGTACTACGCAGCCAAGTATGCAGGCCGTGGTCCCAACCTATTGGGAACAACT
CTGGAGGAGAAGGCAGTTGTCGACCAGTGGCTGGAAGTGGAAGGCCACAACTTCAACGACCTGGTTTA
CACTCTGGTGCTCCAGCTTCTTGTCCTGCCACGGATGGGTCAACGTGGCGACGTTGCATTAATCAATGCA
TGTGAGGAGAAGCTGGAAAAGGTGTTTGATGTTTATGAGGAAAGATTGTCAAAAAGCAAATATCTTGCT
GGAGAAACTTTCACTCTGGCTGATCTGAGCCATCTTCCTGGGATTAGGTATCTGATTGATGAAGCTAAAC
TGGGGCATTTGGTGACTGAGAGGAAGAAAGTGAATGCATGGTGGGAGGACATATCCAACAGGCCTTCT
TGGAAGAAACTAATGCAGCTTGCTAGTGAC

>OG170_DN34207
AAAGAGAAAATGGGCGTGAAAGTGCATGGTCTTGCTCTCTCGACATGCACATCTCGTGTCTTAACCATTC
TCCATGAAAAAGGCCTTGATTTTGAGCTCCTCCCTGTTAACCTTCTTGCAGGAGAACACAAGCAGCCCCA
TTTCCTCGCCAAAAATCCCTTTGGACAGATTCCAGTGCTTGAAGATGGTGATCTCACTCTTTTTGAGTCAA
GAGCTATCACTGCATATGTGGCTGAGAAATTCAAGGAAAATGGTGTTGATCTGATCAGGCACAACAACC
TCAATGAGGCAGCTCTGGTCAAGGTCTGGACAGAAGTGGAGTCCCAGAGTTTCCACCCTGCAATCTCTC
CCATTGTCTTCCAACATATCATCGTCCCCATGCGCGGACAAACACCGGATCAGGCCGACATCGACGCCAA

CTTGGAGAAGCTCGCGAAAGTTCTGGACGTGTACGAGGCCAGGCTGAGTCGCACCAAGTACCTGGCTG
GTGATTTCTACAGCCTTGCTGATCTCCACCACCTTCCCTACACTTACTACTTCATGAAGACTCCGTGGGCG
TCGTTGGTGAATGAACGTCCTCACGTTAAGGCTTGGTGGGAGGACATTTCATCTAGGCCTGCTTTCAAGA
AGGTGGCCGAGGCTATGACCTTGGGACAAAAG

OG517_house_keeping_gene
>OG517_DN6344
ATGGATTCCCGTACAGCCAGGATTTGGATTCCGGTGTTGTTTTTGAGCTGTGCGAGCACCCATTTCTGCT
CTTACGCCGCCGTGGTCGAAGACGACTTGAGGTGCCTTCGGGGGCTGAATCAGTCGCTGCATGATCCGC
TCGAGAAGCTGGCGTCTTGGGATTTCGGGAACACCACCGTAGGAGTCGTCTGCAAGTTCGTCGGCGTCA
CCTGCTGGAACGATAAGGAGAATCGGATCGTCAAACTGGAGCTCCGAGAGATGGAGCTCACCGGCGCG
GTTCCCAAGGAGATAGAGTACTGCGGCAGCCTCACGAGTCTGGATCTCGGCAGCAACGATATTTCCGGG
TCGATTCCGCCGGATATTTGCGATTGGTTGCCCTTTTTGGTGACCCTGGATTTGTCCGGGAACGACTTCTC
CGGCCCGATCCCGCCGGGCCTCCAGCACTGTAAGTATCTGAACAATCTGATCCTTTCAGATAATAAGCTT
ACGGGTCCGATTCCCTATGAATTCTCTAGCTTGGGTAGGCTTAAGAGGTTTTCGGTGGCGAATAATAAGT
TGAAGGGAGCTATCCCCTCGTTTCTCGACGATTTTGATAAGGCGGATTTTGCCGGGAACAGCGGGCTTT
GCGGCGGGCCTCTCGGGTCCAAGTGTGGTGGCTTGAGCAAGAAGAGTCTTGCTATTATAATTGCTGCCG
GAGTTTTTGGCGCGGCTGCGTCTTTGCTGGCGGCTTTGGGGCTGTGGTGGTGGTACCATTTGAGGCTGA
GTAAGCAGCGGAGGACGGGAGGCTTTGGTGTTGCGAGGGAAGATTGGGCTGAAAAGTTGAGGGCTCA
TAGGCTTACTCAAGTTTCCTTGTTTCAGAAGCCACTTGTGAAGGTTAAGTTGGCGGACTTGATGGCGGCT
ACCAATAATTTTAGCCCCGAAAATGAGATCATTTCGACTAGGACGGGGACTACTTACAAGGCCCTGCTG
CCTGATGGGTCGGCGCTGGCCATTAAGCGGCTTAGTGCTTGTAAGCTCGCTGAGAAGCAGTTCCGGTTG
GAGATGAACCGGTTAGGACAGCTTAGGCATCCGAATTTGACCCCCCTTTTGGGTTTCTGCATTGTGGAG
GAGGAAAAGCTTTTGGTATATAAGTACTTGTCGAGTGGGACTTTGCATTCGTTGTTGCACGGAAGTGGT
GAGGAATTGGATTGGCCGACTAGGTTTAGGATCGGTTTGGGTGCTGCCAGGGGACTTGCTTGGCTTCAC
CACGGTTGCCAACCTCCGATTATGCATCAGAACATATGCTCCAATGTGATCCTCCTCGACGAGGATTTTG
ATGCTAGGATAATGGATTTTGGATTGGCAACGCTCACGGCTTCTGATCCTAAGGAGAGCAGTTTCGTCA
ACGGAGACTTGGGAGAGCTCGGTTATGTAGCTCCAGAGTATCCGAGTACTCTGGTTGCTTCACTGAAAG
GGGACGTTTATGGACTTGGAATTGTGCTTCTGGAGCTGGCGACCGGGCAAAAGCCTCTCGAAGTCAGCA
CGGCTGATGAAGGTTATAAGGGCAATGTTGTGGATTGGGTGAATCATCTATATAATTCTGGTCGAACCA
AGGATGCCATCGATAAAGCTCTCATCGGAAAAGGGCATGACGAGGAAATTTTGCAGTTTCTGAAGATTG
CTAGTAGTTGTGTGATTTCTCGGCCTAAGGACAGGTGGTCTATGTACCAGGTTTACCATGCATTGAAGAG
TATGAGCAAAGA<CCACAGTTTCAGGGAACAAGACGACGAGTTTCCATTGCTCTTTCGCAAGCCAGATA
CTGAA>
