# Supplementary material for: Orchard and storage-dependent metabolic and transcriptional regulation of superficial scald in ‘Packham’s Triumph’ pears
Source: Front Plant Sci. 2026 Aug 27;17:1885958. doi: 10.3389/fpls.2026.1885958 (PMC13558034; doi:10.3389/fpls.2026.1885958)
Supplement: Supplementary file 3 [file DataSheet3.pdf]

```
Supplementary file C-1:
Assembly statistics before post-processing:
################################
## Counts of transcripts, etc.
################################
Total trinity 'genes':  44751
Total trinity transcripts:      117239
Percent GC: 43.99

########################################
Stats based on ALL transcript contigs:
########################################

        Contig N10: 3641
        Contig N20: 2850
        Contig N30: 2381
        Contig N40: 2040
        Contig N50: 1767

        Median contig length: 922
        Average contig: 1190.74
        Total assembled bases: 139600761


#####################################################
## Stats based on ONLY LONGEST ISOFORM per 'GENE':
#####################################################

        Contig N10: 3681
        Contig N20: 2839
        Contig N30: 2342
        Contig N40: 1982
        Contig N50: 1664

        Median contig length: 474
        Average contig: 904.76
        Total assembled bases: 40488696


Assembly statistics post processing:
################################
## Counts of transcripts, etc.
################################
Total trinity 'genes':  21904
Total trinity transcripts:      77488
Percent GC: 46.00

########################################
Stats based on ALL transcript contigs:
########################################

        Contig N10: 3207
        Contig N20: 2448
```

```
        Contig N30: 2004
        Contig N40: 1680
        Contig N50: 1440

        Median contig length: 891
        Average contig: 1113.84
        Total assembled bases: 86309334


#####################################################
## Stats based on ONLY LONGEST ISOFORM per 'GENE':
#####################################################

        Contig N10: 3336
        Contig N20: 2541
        Contig N30: 2061
        Contig N40: 1719
        Contig N50: 1473

        Median contig length: 813
        Average contig: 1074.28
        Total assembled bases: 23531115
```

Supplementary file C-2:
Assembly BUSCO output

```
# BUSCO version is: 5.0.0
# The lineage dataset is: eudicots_odb10 (Creation date: 2020-09-10, number of species: 31, number of BUSCOs: 2326)
# Summarized benchmarking in BUSCO notation for file /host_mount/Pt_Trinity_cleaned.cds.fasta
# BUSCO was run in mode: transcriptome

        ***** Results: *****

        C:88.0%[S:35.2%,D:52.8%],F:2.6%,M:9.4%,n:2326
        2047    Complete BUSCOs (C)
        819     Complete and single-copy BUSCOs (S)
        1228    Complete and duplicated BUSCOs (D)
        60      Fragmented BUSCOs (F)
        219     Missing BUSCOs (M)
        2326    Total BUSCO groups searched
```
